# ^1^H Nuclear Magnetic Resonance-Based Targeted and Untargeted Metabolomics Profiling of Retail Samples of Cuachalalate (*Amphipterygium adstringens*)

**DOI:** 10.3390/molecules30102185

**Published:** 2025-05-16

**Authors:** Erick Alejandro Herrera-Jurado, Estefanía De Jesús Terán-Sánchez, José Iván Serrano-Contreras, L. Gerardo Zepeda-Vallejo

**Affiliations:** 1 Departamento de Química Orgánica, Escuela Nacional de Ciencias Biológicas, Instituto Politécnico Nacional, Prolongación de Carpio y Plan de Ayala s/n, Col. Santo Tomas., Delegación Miguel Hidalgo, Mexico City C.P. 11340, Mexico; erick.cqb@gmail.com (E.A.H.-J.); estefaniateran92@gmail.com (E.D.J.T.-S.); 2 Department of Metabolism, Digestion and Reproduction, Section of Biomolecular Medicine, Faculty of Medicine, Imperial College London, South Kensington Campus, London SW7 2AZ, UK; j.serrano-contreras@imperial.ac.uk

**Keywords:** *Amphipterygium adstringens*, cuachalalate, ^1^H NMR, metabolomics profiling, 3α-hydroxy-masticadienoic acid, anacardic acids

## Abstract

*Amphipterygium adstringens* (cuachalalate) is a medicinal plant widely used in traditional Mexican medicine for its anti-inflammatory, gastroprotective, and antimicrobial properties. In this study, we applied qualitative and quantitative NMR-based metabolomics profiling, combined with multivariate statistical analyses, including Principal Component Analysis (PCA), Partial Least Squares Discriminant Analysis (PLS-DA), and S-plots, to evaluate the chemical composition and authenticity of *A. adstringens* samples collected from different commercial sources sold in Mexico City. Metabolomic profiles in organic and aqueous extracts revealed highly similar spectral patterns among all collected samples, supporting the consistency of commercially available *A. adstringens* in Mexico. The presence of 3α-hydroxy-masticadienoic acid (3α-HMDA) and anacardic acids, biomarkers of the genus, was confirmed by ^1^H NMR in hexane extracts; in the aqueous extract they were not observed with the same analytical platform. These findings suggest that the traditional infusion method may not effectively extract the above-mentioned key bioactive compounds. This approach enhances quality control and ensures the reliability of *A. adstringens* products in the commercial market.

## 1. Introduction

The use of medicinal plants is a deeply rooted tradition in different Mexican cultures that has remained active throughout its history [1]. There are reports of approximately 23,400 vascular plants in Mexico of which 3000 have a medicinal effect [2]. It is worth highlighting the antimicrobial effect of a large number of these plants [3,4]. It is well known that *Helicobacter pylori* is the etiologic agent of peptic ulcer, and it is also associated with gastric cancer [5]. A wide variety of plants have been studied against this microorganism, finding that methanolic extracts of *Persea americana*, *Annona cherimola, Guaiacum coulteri,* and *Moussonia deppeana* are the most effective [6]. Recently, it has been demonstrated that *Artemisia ludoviciana* subsp. *mexicana* shows significant activity against *Helicobacter pylori*, whose aqueous extract showed MIC of 250 µg/mL, as well as bactericidal effect. Moreover, a gastroprotective activity of up to 69.8 ± 3.8% at 300 mg/kg (oral administration), and anti-inflammatory effects of 47.6 ± 12.4% (oral) and 38.8 ± 10.2% (topical) were shown in murine models [7]. It has also been shown that *Amphipterygium adstringens* (synonymies *Juliania adstringens*, known in folk medicine as cuachalalate), exhibits activity against *H. pylori*, being a plant widely distributed in the Mexican territory. It has been estimated that 57.5 tons of its bark are harvested annually, with the main collection areas being the state of Morelos, the Mixteca Poblana, and the northern region of the Balsas Basin, located in south-central Mexico [8]. Cuachalalate is a 10 m tall tree with a twisted trunk and scales on the bark; its leaves are opaque green on the front and grayish on the back, and are grouped at the tips of the branches [9]. The distribution of this medicinal tree is found in the states of Sinaloa, Nayarit, Jalisco, Colima, Michoacán, Morelos, Guerrero, Oaxaca, and Chiapas [8].

The bark of this tree has been used in traditional Mexican medicine to treat skin lesions and gastric ulcers and as a cicatrizing promoter. Its anti-inflammatory and proangiogenic properties are attributed to 3α-hydroxymasticadienoic acid (3α-HMDA) and anacardic acids [10]. The latter shows activity against *H. pylori* (MIC of 10 μg/mL) [11]. It is also known that 3α-HMDA inhibits cell growth in human cancer lines and also stimulates nitric acid production in macrophages. Recently, this metabolite has been shown to induce the maturation of dendritic cells that promote the activation of T lymphocytes [12]. The biological activity of pyrolysis-derived oils of *Amphipterygium adstringens* has recently been investigated, revealing significant inhibitory effects on the production of IL (Interleukin)-8. This suggests their potential utility in researching treatments for dermatological diseases influenced by IL-17, such as psoriasis. It is noteworthy that one of the oils demonstrated an inhibitory effect at 15 µg/mL, comparable to dexamethasone in IL-8 [13].

In 1962, catechol and a steroidal saponin were found in the n-butanolic extract of the bark of cuachalalate [14]. Also, a phytochemical study of the plant by gas chromatography coupled to mass spectrometry (GM-MS) revealed the presence of fatty acids, such as myristic, pentadecanoic, palmitic, linoleic, oleic, hexadecanoic, and stearic acids, as well as the monoterpene α-terpineol and the phenol cardanol [15]. Makino and colleagues investigated methanolic extracts, detecting the presence of nine triterpenes, including schinol, oleanonic acid, 3a-hydroxy-11a,12a-epoxy-oleanane-28,13b-olide, 3b-hydroxy-11a,12a-epoxy-oleanane-28,13b-olide, β-sitosterol, and ocotillone [16]. Recent studies indicate that among the main components found in the bark of cuachalalate are 3-epioleanolic acid, β-sitosterol, masticadienoic acid, and 3α-HMDA [12].

The combination of bioactive molecules in synergy with well-established drugs, such as cisplatin (cis-diaminedichloroplatinum (ll) or CDDP), has been investigated in prostate cancer PC3 and colon cancer HCT116 cell lines. Two of the most important components of the bark, masticadienoic acid (MDA) and 3α-HMDA, were used for this purpose. In both cell lines, an increase in cytotoxic effects was observed with all combinations of MDA and CDDP, as assessed by the sulforhodamine B assay [17].

The Federal Commission for the Prevention of Health Risks (COFEPRIS, Comisión Federal para la Prevención de Riesgos Sanitarios-México), through the herbal medicine section of its official journal, authorizes the use of cuachalalate as an ingredient in herbal remedies [18], in which it is usually consumed as an infusion from small pieces of bark or as a powder in tablets or pills. This plant is described in the Herbal Pharmacopoeia of the United Mexican States (FEUM, Farmacopea Herbolaria de los Estados Unidos Mexicanos), in which the change of genus *Amphipterygium* to *Juliania* is mentioned [19]. It also describes the methodology to perform an identity assay based on the presence of its biomarker molecule, 3α-HMDA, using liquid chromatography coupled to a UV/VIS detector at 215 nm.

Due to the medicinal and commercial importance of cuachalalate bark, it is important to demonstrate the presence of the metabolites responsible for its biological activity in commercial samples, as it is well known that herbal products can be subject to adulteration by morphologically similar species that may not have the active ingredients [20]. In this context, the present work describes the metabolomics profiling of aqueous and organic extracts of 10 samples of cuachalalate collected from different stores in Mexico, thus providing a practical protocol to assess its authenticity and safety of consumption.

## 2. Results and Discussion

### 2.1. Hexane Extracts as Criterion to Autentify the Samples Studied

**Figure 1:**
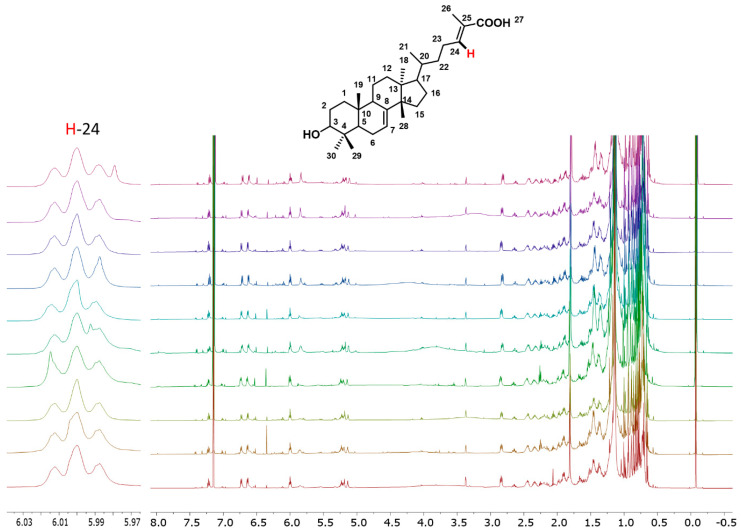
^1^H NMR spectra (600.18 MHz, CDCl_3_) of trichloroethylene extracts of cuachalalate. Characteristic triplet signal at 6.0 ppm belongs to H-24 of 3α-HMDA.

A total of ten retail samples were collected from diverse markets and herbalists in Mexico City (Table S1), thereby encompassing potential variability attributable to different origins, collection periods, or storage conditions. Afterward, it became essential to perform a plant identity test in accordance with the guidelines of the Mexican Herbal Pharmacopoeia. This establishes the presence of the 3α-HMDA biomarker in hexane extracts. Thus, characteristic signals of this metabolite were identified from the hexane extracts, primarily the triplet signal of H-24 at approximately δ 6.0 (Figure 1). To explore potential improvements in extraction yield, additional trials were conducted using different solvents including trichloroethylene, dichloromethane, hexane, and acetonitrile even though hexane was the standard. Among the tested solvents, trichloroethylene proved to be the most effective in enhancing extraction efficiency (Figure S1). The presence of this biomarker was used as the first criterion for further analysis of each studied sample. Chromatographic separation was then performed using hexane-ethyl acetate as eluent, starting with pure hexane and then continuing with a ratio of 9:1, 8:2, 7:3, and finally ending with pure ethyl acetate. This allowed for the separation of the aforementioned biomarker, as well as a mixture of anacardic acids, other targeted bioactive molecules present in authentic cuachalalate samples. In Figure S2 are shown the ^1^H and ^13^C NMR parameter assignments of 3α-HMDA. Full assignment of the ^1^H and ^13^C NMR spectra of this biomarker was achieved by performing a series of 1D and 2D NMR experiments shown in Figures S3–S7.

### 2.2. Metabolomic Profiling of the Aqueous Extracts of Amphipterygium adstringens

**Figure 2:**
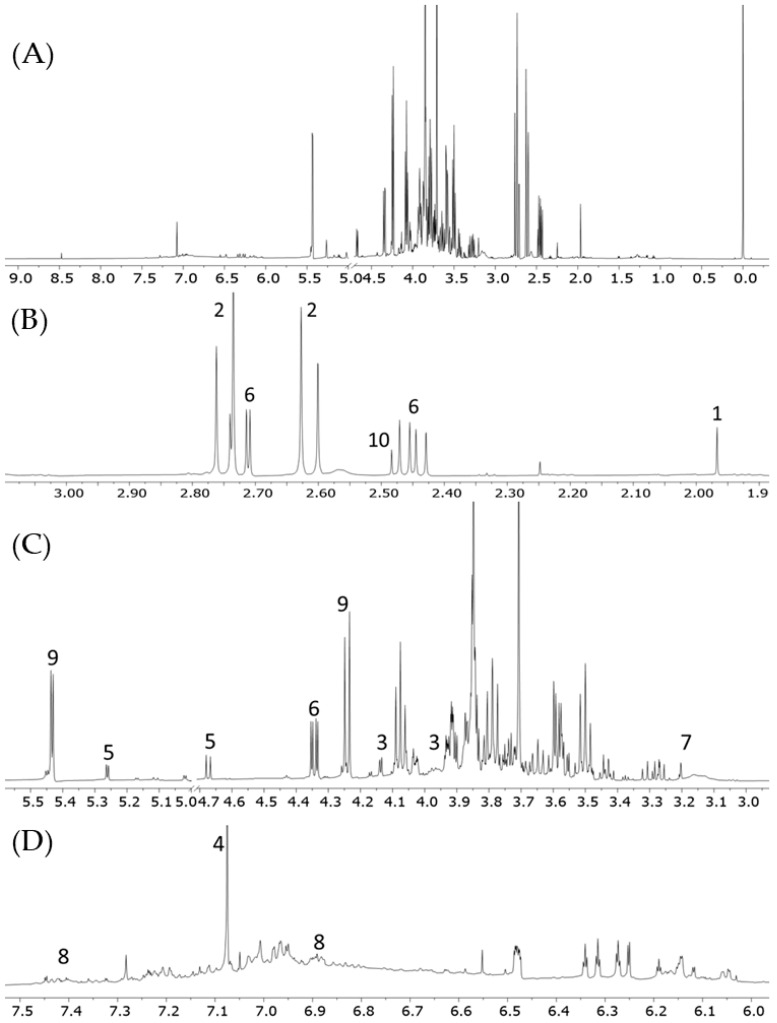
^1^H NMR spectrum (600.18 MHz, H_2_O/D_2_O) of a pooled sample (SMP11) of cuachalalate shown in several expanded sections: (**A**) *δ* −0.25–9.18; (**B**) *δ* 1.88–3.78; (**C**) δ 3.40–5.57; (**D**) *δ* 5.60–7.53. Label numbers specify the signals belonging to the following metabolites: acetic acid, 1; citric acid, 2; fructose, 3; gallic acid, 4; glucose, 5; malic acid, 6; malonic acid, 7; protocatechuic acid, 8; sucrose, 9, succinic acid, 10.

Once the identity of the ten cuachalalate samples was confirmed, aqueous extracts were obtained from the ten samples (SMP1–SMP10) simulating the usual conditions of tea preparation for consumption (Section 3.2). Water was heated to 80 °C, and the samples were left to stand for 5 min before continuing with the aforementioned procedure. A total of a hundred and ten ^1^H NMR spectra were obtained, on which statistical analyses were performed. All ^1^H NMR spectra were subjected to baseline and phase correction, as well as being normalized and referenced to the TSP signal at 0 ppm, which was also set to 1 on the Y-axis. Afterward, they were divided into small regions of 0.04 ppm, called bins, which denote the number of variables for statistical analysis. This procedure produced a variable matrix in which each variable represents a specific chemical shift region (bin), with its value corresponding to the area under the curve within that bin. These variables reflect the chemical profiles of the metabolites in the analyzed samples and are directly related to the chemical profiles of the metabolites present in that samples. A quality control (QC) sample was prepared to obtain the ^1^H NMR spectrum (Figure 2) to which the signals of the identified metabolites were assigned. By averaging the concentration of metabolites present in all samples, the QC sample allows to observe the signals of metabolites that are absent or present at low concentrations in some individual samples. Also, the ^1^H NMR spectrum of the QC sample showed proper alignment with the rest of the samples, providing elements of confidence during preprocessing. Finally, SMP11 was included in the exploratory PCA and PLS-DA (Figure S8), where it appeared tightly clustered at the center of the sample groups without overlapping, supporting reproducibility and instrumental stability. Table S2 shows the most abundant identified metabolites and Figure S9 shows the bar graphs of metabolites and their concentration in each sample. The distribution of metabolites varied in terms of their presence and concentration in each of the samples analyzed. Glucose, sucrose, fructose, and gallic, malic, citric, succinic, and acetic acids were found in all samples from SMP1–SMP10, as expected for aqueous plant extracts. Another variable metabolite found was protocatechuic acid, whose signals were clearly identified in the ^1^H NMR spectrum of sample SMP1 (Figure S10a,b). The signals of this compound were barely perceptible in the ^1^H NMR spectra of the other samples due to its low concentration (Figure S10c).

Vanillic acid was found only in SMP1 at an average concentration of 0.5374 mM. This metabolite has been used as a flavoring agent and preservative. It is known for its pharmacological properties such as antioxidant, anti-inflammatory, immuno-stimulating, neuroprotective, and hepatoprotective effects [21].

It is interesting to note that studies have been conducted on the activity of vanillic acid in ulcerative colitis, a property similar to that presumed for the anacardic acids present in this plant, which also serve as gender biomarkers [22]. It can be observed that in SMP1 the concentrations of citrate, succinate, and malate are significantly lower than in the retail samples (Figure S9). These metabolites are key intermediates in the Krebs cycle, and their concentrations have been shown to vary depending on environmental conditions, indicating that they can change over time [23]. It is important to emphasize that the objective of this study was to analyze the product as it reaches consumers; therefore, no control was exerted over the previous steps, from plant collection to shelf life.

### 2.3. Multivariate Statistical Analyses

#### 2.3.1. Aqueous Extracts—Untargeted Metabolomic Profiles of Cuachalalate Samples

Figure 2A shows the high predominance of signals belonging to sugars in the ^1^H NMR spectrum of aqueous extracts. This situation could lead to a bias in the statistical model as intense sugar signals can mask less abundant metabolites with significant variance. In this sense, as a preliminary step to the processing, two data matrices were generated from the ^1^H NMR spectra of the aqueous extracts of cuachalalate. The first matrix included the sugar signals (Matrix Including Sugars, MIS), and in the second matrix the corresponding signals were excluded (Matrix Excluding Sugars, MES). Thus, multivariate statistical analysis comprising Principal Component Analysis (PCA) and Partial Least-Squares Discriminant Analysis (PLS-DA) were performed in both matrices to observe the trend of the samples, which were obtained with Pareto scaling using five components.

In Figure S11A, the graphical results of the PCA performed on MIS samples can be observed, from which R^2^ (0.937) and Q^2^ (0.898) values were obtained. The analysis yielded variance values of 46%, 25%, and 12% for the first (PC1), the second (PC2), and the third component (PC3), respectively. These results suggest that a substantial portion of the variability in the dataset is captured by the first few components, with PC1 being particularly dominant in summarizing the main variation in the data. On the other hand, PCA of the MES yielded slightly lower values, with R^2^ = 0.931 and Q^2^ = 0.846, and the variance explained by PC1, PC2, and PC3 was distributed as 41%, 23%, and 17%, respectively (Figure S11B). Although the MES shows slightly lower explanatory and predictive performance compared to the MIS, it still provides robust insights into the dataset’s structure. The increased contribution of PC3 in the MES suggests that it captures more variability in higher-order components, which could be relevant for identifying subtle or secondary patterns in the data.

**Figure 3:**
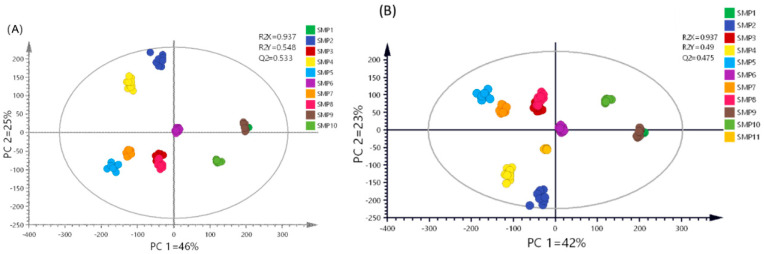
Score plots of PLS-DA of the aqueous matrices (**A**) including sugars (MIS) and (**B**) excluding sugars (MES) of the ten samples studied (SMP1–SMP10).

A multivariate statistical analysis was then performed in MIS samples by using Partial Least Squares Discriminant Analysis (PLS-DA), which allowed grouping by study classes (Figure 3A). R^2^X, R^2^Y, and Q^2^ values of 0.937, 0.548, and 0.533, respectively, were obtained. Although the model excels in explaining the variance in the predictor variable (X), the fit to the response variables (Y) and the predictor performance (Q^2^) are moderately good. PLS-DA of the MES yielded R^2^X = 0.93, R^2^Y = 0.536, and Q^2^ = 0.507 (Figure 3B) demonstrating good performance; the high R^2^X value indicates that most of the variance in the input data is explained. The R^2^Y values for both matrices reflect the capacity to explain the variance in the response variable, and the Q^2^ values confirm the predictive reliability of the models. In both cases the validation was performed using 200 permutations (Figure S12).

Notably, the MIS outperformed the MES in all three metrics, albeit by a narrow margin, suggesting a slightly better ability to distinguish the groups within the dataset. This behavior can be explained by the fact that the elimination of sugars leads to the elimination of discriminating variables, reflecting that sugar largely drives the differentiation between samples. The multivariate analysis reveals distinct metabolic drivers in each principal component. In the MIS, PC1 is predominantly governed by sugar signals, while PC2 shows significant contributions from non-sugar discriminating metabolites (Figure 3A). Remarkably, the MES loading plot demonstrates clear class separation, indicating that non-sugar metabolites effectively preserve the sample differentiation patterns (Figure 3B). This suggests that the underlying variability factors (geographical origin, collection period, storage conditions, etc.) are captured by these alternative metabolic markers despite sugar removal. It can be stated that sugars are important but not exclusive for discrimination in cuachalalate samples.

**Figure 4:**
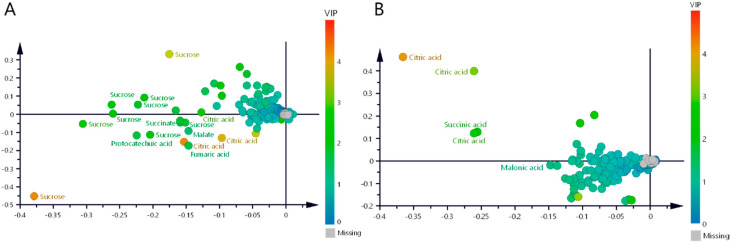
Loading scatter plots of (**A**) matrix including sugars (MIS) and (**B**) matrix excluding sugars (MES).

Once the score plots showed the variability between MIS and MES samples, loading scatter plots were generated in both matrices to determine which metabolites caused the separation between the samples. Figure 4A,B show the loading scatter plots of the MIS and MES matrices, respectively. These plots show the scale of the VIP values and reveal the variables that contribute most to the predictive model. For example, the MIS loading scatter plot confirms that sucrose is one of the most discriminating metabolites among the classes in this matrix (VIP = 4.21), followed by citrate (VIP = 4.2). Other metabolites such as fumarate, succinate, and malate have VIP values for a given bin of 1.63, 1.61, and 1.34, respectively, which also significantly contribute to the predictive model or discrimination between classes. The loading plot analysis of the MES matrix identified citrate as the predominant discriminatory metabolite, showing the highest variable importance (VIP = 4.07). This was followed by succinate (VIP = 2.44) and malonate (VIP = 1.21) in descending order of contribution to the predictive model. Notably, this analysis uncovered malonate as a newly significant discriminatory metabolite that was not detected in the parallel analysis of the MIS matrix, suggesting its potential role as a distinguishing feature in the sugar-free metabolic profile.

**Figure 5:**
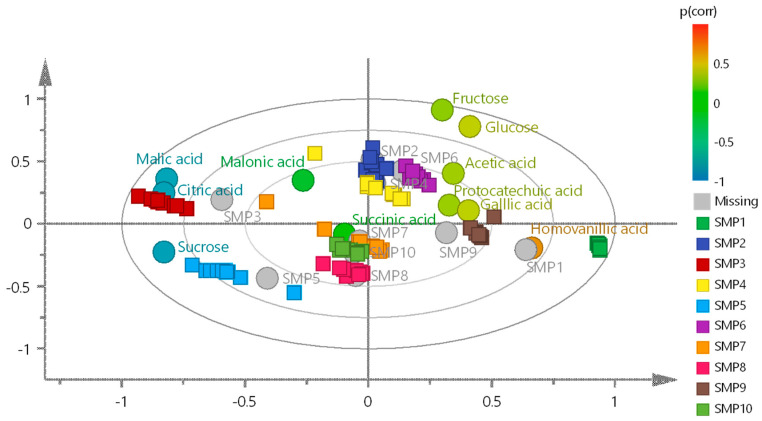
Biplot showing the correlation between metabolites and samples SMP1–SMP10.

A biplot was created to observe the correlation between variables and their classes (Figure 5). Individual samples (SMP1 to SMP10) display a heterogeneous clustering pattern within the Hotelling’s T^2^ ellipses, suggesting variations in their chemical profiles potentially linked to genetic, environmental, or experimental factors. Samples SMP2, SMP4, and SMP6 show high metabolic similarity, while SMP7, SMP8, and SMP10 are located near the origin, reflecting lower variation relative to the overall group because they also have comparable profiles. Notably, SMP3, SMP5, and SMP1 lie within the 95% confidence ellipse, indicating significant metabolic divergence from the other samples. SMP5 shows the strongest negative correlation with sucrose, while SMP3 shows analogous behavior with citric and malic acids. In contrast, SMP1 is positively correlated with homovanillic acid, a key discriminating metabolite for this sample. The corresponding PLS-DA model was also validated with 200 permutations and five components (Figure S13). Metabolites further from the origin, such as fructose, glucose, citric acid, and malic acid, play significant roles in separating the samples within the principal component space. Fructose and glucose, associated with the positive axis of the first principal component, appear linked to metabolic responses involving simple sugar storage or consumption. In contrast, citric and malic acids, located along the negative axis, are likely associated with primary metabolic pathways such as the Krebs cycle. Gallic acid, situated along the positive axis of the second principal component, stands out for its potential involvement in antioxidant mechanisms, while succinic acid and acetic acid may play important roles in energy and secondary metabolism.

These results emphasize the utility of multivariate analysis (PCA and PLS-DA) in identifying metabolic patterns and biomarkers associated with cuachalalate samples. However, validating these findings through additional statistical tests and functional studies is essential to uncover underlying mechanisms. Furthermore, the central positioning of SMP11 in the statistical space of PLS-DA (Figure S8) reinforces its importance as a comprehensive reference for comparing the observed variability across individual samples, solidifying its use in future metabolic studies. This approach provides valuable insights into the differences in active metabolic pathways and may serve as a foundation for pharmacological or biotechnological applications of cuachalalate.

#### 2.3.2. Organic Extract—Targeting the Analysis Through 3α-HMDA and Anacardic Acids

In the process of organic extraction trichloroethylene was employed, resulting in the acquisition of a total of fifty ^1^H NMR spectra. Subsequent statistical analysis, specifically PCA, revealed R^2^ and Q^2^ values of 0.926 and 0.893, respectively, including the five components containing the total of the explained variance (Figure S14). In this sense, the first principal component (PC1) accounted for 64%, while the second (PC2) explained 11% of the total variance.

Similarly to the procedure followed in aqueous extraction, PLS-DA was performed to assess class discrimination. The analysis yielded values of R^2^X = 0.92, R^2^Y = 0.509, and Q^2^ = 0.428. The examination of the loading plot and S-plot identified variables corresponding to the chemical shifts associated with the biomarker 3α-HMDA, being the most discriminant metabolite in organic extract, as observed in Figure S14.

**Figure 6:**
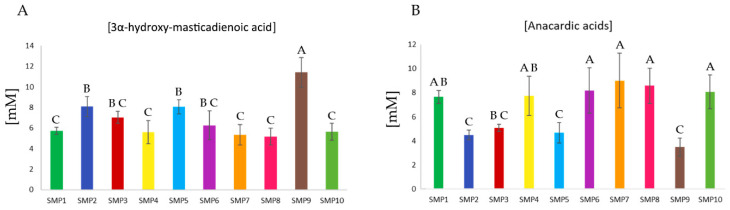
Variation in the relative concentrations of (**A**) 3α-HMDA and (**B**) anacardic acids in samples SMP1–SMP10. Capital letters in the top of each column indicate significant mean differences (Tukey’s HSD test, *p* < 0.05).

Given the significance of the bioactive molecules identified and verified through purification in this study, the relative concentrations of the biomarkers 3α-HMDA and anacardic acids were measured. The analysis yielded more consistent levels of 3α-HMDA across the samples, whereas the concentration of anacardic acids exhibits greater variability. The relative concentration was determined using the formula [24]:

$$Mx=Mst\frac{Ax}{Ast}\times \frac{Nst}{Nx}\times \frac{MWx}{MWst}$$

where *Mx* is the unknown mass of the targeted analyte, and *M_ST_* is the mass of the internal standard; *A_X_* and *A_ST_* are the integral areas for the selected signals; *MW_X_* and *MW_ST_* are the molecular weights of the targeted analyte and the standard; *N_X_* and *N_ST_* are the number of protons generating the integral signals, respectively. The triple signal appearing at 6.00 ppm belonging to H-24 of 3α-HMDA, as well as the double signal belonging to H-5 of anacardic acids (Figure S15), were used to determine their relative quantification. Using the internal standard, hexamethyldisilane, at a known concentration of 1 mM as a reference, the relative concentrations for both studied species are shown in the following bar graphs (Figure 6).

As can be observed in the graphs, the concentrations for 3α-HMDA range from 5.16 mM in SMP8 to 11.4 mM in sample SMP9. Regarding the anacardic acids, the concentrations exhibit a range from 3.4 mM in SMP9 to 9.01 mM in SMP7. The variation in metabolite concentration, even within the same plant and using the same aliquot, is variable. This may be due to the season of collection, the plant’s (sexual) gender, or even storage time [25]. Variations in metabolites such as amino acids, flavonols, or catechins have been observed due to this effect [26].

In contrast with trichloroethylene extract, 3α-HMDA and anacardic acids were not detected in the aqueous extract (Figure 2), as their concentration may be too low to be detected by NMR. In consonance with our results, it was recently described that 3α-HMDA and anacardic acids were detected in hexane extracts but not in aqueous extracts [27]; instead, saponins were identified in the last extract. It could be hypothesized that triterpenoid structures, such as 3α-HMDA, are present as glycosylated derivatives at low concentrations. The interest in this type of compounds remains relevant, as the extraction of sarsapogenins using supercritical CO_2_ was recently described [28]. It should be mentioned that the aqueous extract of *Juliana adstringens* (*Amphipterygium adstringens*) possesses inhibitory potential against microorganisms such as *Streptococcus mutans* and *Porphyromonas gingivalis*, showing Minimum Inhibitory Concentration (MIC) ranging from 67.5 to 500 µg/mL [3].

## 3. Materials and Methods

### 3.1. Collection and Preparation of Botanical Material

A total of 10 retail samples were collected in Mexico City in 2022 from various markets and stores specializing in herbal remedies (see Table S1). Samples were ground to powder consistency using a mechanical grinder and stored in the dark in zip-lock bags until extraction. Each sample was labeled as SMP1 to SMP11, where SMP1 corresponds to a sample collected directly from an authenticated *Amphipterygium adstringens* tree (La Capilla, Arteaga, Michoacán; Mexico; 18°20′09.3″ N, 102°17′13.3″ W), and SMP2 to SMP10 are samples obtained from different points of sale (Table S2). In addition, the label SMP11 denotes a quality control sample (QC) pool, which is a mixture of equal parts of SMP1 to SMP10 (0.5 g each). This quality control sample was processed in the same way as the rest of the samples. The QC sample was prepared to obtain the ^1^H NMR spectrum (Figure 2) to which the signals of the identified metabolites were assigned. By averaging the concentration of metabolites present in all samples, the QC sample allowed to observe the signals of metabolites that are absent or present at low concentrations in some individual samples. Also, the ^1^H NMR spectrum of the QC sample showed proper alignment with the rest of the samples, providing elements of confidence during preprocessing. Finally, SMP11 was included in the exploratory PCA and PLS-DA (Figure S8), where it appeared tightly clustered at the center of the sample groups without overlapping, supporting reproducibility and instrumental stability.

### 3.2. Obtaining Aqueous Extract from Cuachalalate Samples

An amount of 1 g of each powdered sample (SMP1 to SMP11) was poured into 10 milliliters of high-performance liquid chromatography (HPLC) grade water (Fermont, Productos Químicos Monterrey, SA de CV, Mexico) previously heated at 80 °C and allowed to stand for 5 min. This process was repeated ten times for each sample (SMP1–SMP11). The resulting extracts were filtered through a hydrophilic filter of 0.22 μm. Once the samples were filtered, 400 µL of each sample was transferred to an Eppendorf tube containing 140 µL of phosphate buffer at pH 5 and 60 µL of a standard solution of 3–trimethylsilylpropionic acid-d4 sodium salt (TSP, 10 mM) with ethylenediaminetetraacetic acid (EDTA, 10 mM) to obtain a final concentration of 1 mM of TSP and EDTA. Finally, the sample was centrifuged for 5 min at 12,000 rpm, after which 600 µL was transferred to an NMR tube to acquire the corresponding ^1^H NMR spectra.

### 3.3. Obtaining Organic Extracts from Cuachalalate

To obtain the organic extract of cuachalalate, 1 g was extracted in a conical tube with 14 mL of trichloroethylene by sonication for 15 min at 40 °C. Then, 2 mL were filtered and evaporated to dryness at 35 °C. The dried extracts were re-suspended in a solution of 540 μL of deuterated chloroform (CDCl_3_) and 60 µL of 10 mM hexamethyldisilane (HMDS), used as internal reference. For extraction trials, the solvents trichloroethylene, dichloromethane, hexane, and acetonitrile were used, all of them HPLC grade (J.T. Baker, Avantor, Mexico). As shown in Figure S1, trichloroethylene was the most effective solvent for selective extraction.

To obtain the bioactive compounds 3α-HMDA and anacardic acids, an isocratic column chromatography was performed using a 3:2 mixture of hexane/ethyl acetate as eluent. The separation column used had a height of 40 cm and a diameter of 5 cm. Silica gel was used with gravity-based separation (300–430 mesh, Natland International Corporation). A total of 106 fractions were collected (10 mL/fraction). The biomarker 3α-HMDA was collected from fractions 89–106, as revealed by thin layer chromatography (TLC). The structure of 3α-HMDA is fully supported by its NMR spectra shown in Figures S10–S14, whose ^1^H and ^13^C NMR parameters are described in Figure S2. It is worth mentioning that anacardic acids were isolated as a complex mixture resulting from different lengths of the aliphatic chains attached to the aromatic ring (Figure S15).

### 3.4. Acquisition of the ^1^H NMR Spectra

#### 3.4.1. ^1^H NMR of Aqueous Samples

^1^H NMR spectra of aqueous extracts were obtained on a Bruker Avance III 600 MHz spectrometer (14.1 T, BioSpin, Rheinstetten, Germany) equipped with a 5 mm broad band observe (BBO) probehead (PA BBO 600S3 BBF-H-D-05 Z SP) using the NOESYPR1D pulse sequence for water suppression. Aqueous extracts of cuachalalate were measured at 25.0 ± 0.1 °C. Acquisition parameters were set as follows: scan number = 256, FID size = 32 K, spectral width = 16.00 ppm, receiver gain = 49.7, acquisition time =1.70 s, relaxation time = 5 s. The range of linewidth was 0.8 to 1.4 Hz. A total of 11 classes (including SMP11) yielded an array of 110 ^1^H NMR spectra.

#### 3.4.2. ^1^H NMR of Organic Samples

^1^H NMR spectra of organic extracts were also obtained on a Bruker 600 AVANCE III HD using the zg30 pulse sequence. Organic extracts of cuachalalate were measured at 25.0 ± 0.1 °C. Acquisition parameters were set as follows: scan number = 64, FID size = 12 K, spectral width = 19.00 ppm, receiver gain = 30.3, acquisition time =2.72 s, relaxation time = 5 s. Employing this set of parameters and implementing a suitable shimming, we were able to attain an average linewidth of 0.8–1.4 Hz. A matrix of fifty ^1^H NMR spectra was obtained.

### 3.5. Metabolites Identification on the ^1^H NMR Spectra

Metabolites identification was performed by consulting available online databases such as HMDB (https://www.hmdb.ca, accessed on 19 November 2024) and NP-MRD (https://np-mrd.org/, accessed on 17 January 2025) and using the software Chenomx Suite ver. 8. Finally, confirmation of the ^1^H NMR signal assignment was achieved by acquiring 2D NMR spectra such as J-resolved, HMBC, COSY, and TOCSY (Figure S16).

### 3.6. ^1^H NMR Data Processing

Each one of the raw FIDs was processed with MestreNova software (version 12.0.2-20910). The baseline and phase were corrected, and the internal standard was calibrated to 0.0 ppm. The integration of the ^1^H NMR spectrum was referenced to the TSP signal. Line broadening apodization was performed at 0.3 Hz. A binning size of 0.04 ppm was used, from 0.2 to 10 ppm. In the case of the aqueous samples, the water suppression region was discarded (4.70–5.00 ppm) before the statistical analysis.

### 3.7. Statistical Analysis

Principal component analysis (PCA) was performed, followed by supervised Partial Least Squares Discriminant Analysis (PLS-DA). The quality of the model was defined by the R^2^X and Q^2^ values. To measure the contribution of the different variables in the model, the variable importance parameter (VIP > 1) was considered. The software SIMCA version 14.1 (Umetrics, Kinnelon, NJ, USA) was used to obtain these assays.

## 4. Conclusions

According to the analysis of cuachalalate samples collected at different points of sale, it was confirmed that all examined samples belong to *Amphipterygium adstringens*, as indicated by the presence of the genus biomarkers 3α-HMDA and anacardic acids. The results demonstrate that all analyzed cuachalalate samples, collected from various locations across Mexico City, exhibit chemical profiles consistent with the reference sample (SMP1). It should be noted that when prepared as a traditional aqueous infusion or tea, the bioactive compounds may be present in concentrations too low to be detected by NMR, unlike what occurs in selective extractions with trichloroethylene. In contrast to HPLC, the only method described to authenticate cuachalalate, NMR does not need derivatization, sample preparation is straightforward, and metabolite identification is often more precise and efficient in raw extracts. Concerning the quality of the retail samples, their metabolomic profiles were highly consistent across all of them, regardless of extraction method (aqueous or organic) or origin. Hexane extracts also exhibited uniform chemical compositions, complying with the Herbal Pharmacopoeia of the United Mexican States. Accordingly, the present results confirm NMR spectroscopy as a rapid, reliable tool for authentication and quality control of commercial cuachalalate (*Amphipterygium adstringens*).

## Data Availability

The data presented in this study are available on request from the corresponding author.

## Supplementary Materials

The following supporting information can be downloaded at: https://www.mdpi.com/article/10.3390/molecules30102185/s1, Figure S1. 1H NMR spectra of the selective extracts of 3a-hidroxymasticadienoic acid (3α-HMDA) obtained from cuachalalate in different organic solvents: (A) Trichloroethylene, (B) Dichloromethane, (C) Hexane, (D) Acetonitrile. Signals intensities are referenced to the signal of 10 mM hexamethyldisilane in CHCl_3_.; Figure S2. ^1^H and ^13^C NMR data of 3α-hydroxymasticadienoic acid (3α-HMDA). The NMR data described below were obtained on a Bruker Avance III 600 MHz spectrometer. The corresponding multiplicities, coupling constants, and assignments were performed by using the series of 1D and 2D NMR experiments shown in Figures S3–S7. The analyzed sample was obtained after separation through a column chromatography packed with 300-400 mesh silica gel, using a 3:2 hexane:ethyl acetate mixture as eluent; Figure S3. ¹H NMR spectrum of the purified biomarker 3α-HMDA; Figure S4. ^13^C NMR spectrum of the purified biomarker 3α-HMDA (CDCl3, 150 MHz); Figure S5. a. ¹H-^13^C HSQC spectrum of the purified biomarker 3α-HMDA (CDCl3, 600 MHz); b. Expanded region of the ¹H-^13^C HSQC spectrum of 3α-HMDA (CDCl_3_, 600 MHz); Figure S6. a. HMBC spectrum of the purified biomarker 3α-HMDA (CDCl_3_, 600 MHz); b. Expanded region of the HMBC spectrum of 3α-HMDA (CDCl_3_, 600 MHz); Figure S7. ¹H-¹H COSY spectrum of the purified biomarker 3α-HMDA (CDCl3, 600 MHz); Figure S8. PCA (A) and PLS-DA (B) score plots of all samples including QC, labeled as SMP11; Figure S9. Bar graphs show the relative concentration of the identified metabolites in the aqueous extract. ANOVA (t-student, *p* = 0.05); Figure S10. a. ^1^H NMR signals of protocatechuic acid identified with Chenomx^®^ in SMP1; b. ^1^H NMR spectrum of the aqueous extract of cuachalalate showing signals of protocatechuic acid in SMP1. The ^1^H NMR signals for aromatic protons (7.3–7.5 ppm) in the theoretical spectrum match very well those of the experimental spectrum; c. ^1^H NMR spectrum of the aqueous extract of cuachalalate (SMP4) showing very weak signals of protocatechuic acid. Similar behavior was observed for samples SMP2, SMP3, SMP5-SMP10. It can be observed that the metabolite signals are negligible due to their low concentration; Figure S11. PCA of aqueous matrix of SMP1-SMP10 (A) including sugars (MIS) and (B) excluding sugars (MES); Figure S12. Validation of the PLS-DA model on MIS (top) and MES (bottom) samples using 200 permutations; Figure S13. Validation of the PLS-DA model on MIS (top) and MES (bottom) samples using 200 permutations; Figure S14. Multivariate statistics on organic extracts. (A) PCA, (B) PLS-DA, (C) Loading plot and (D) S-plot; Figure S15. ^1^H NMR spectrum of anacardic acids isolated from cuachalalate. Expansion region shows in detail signals belonging to aromatic protons (CDCl_3_, 600 MHz); Figure S16. 2D NMR spectra of cuachalalate aqueous extract (H_2_O:D_2_O 90:10, 600 MHz) used to confirm metabolite identification. Once the metabolites present in the aqueous mixture were identified by Chenomx^®^, they were confirmed by the set of spectra shown: J-resolved, allowed accurate determination of chemical shifts and scalar coupling in partially overlapping signals; ^1^H-^1^H COSY, provided connectivity information via scalar coupling; HSQC, allowed assignment of carbons associated with each proton via heteronuclear scalar coupling to one bond; HMBC, was useful for assignment of carbons exhibiting heteronuclear scalar couplings to two and three bonds; Table S1. The samples were obtained at their points of sale, processed to a powdered consistency, and stored in resealable bags in a dry and dark place until analysis; Table S2. Identified metabolites in the aqueous extract of retail samples of cuachalalate.

## References

[1] Mata R., Figueroa M., Navarrete A., Rivero-Cruz I., Kinghorn A., Falk H., Gibbons S., Kobayashi J., Asakawa Y., Liu J.K. (2019). Chemistry and Biology of Selected Mexican Medicinal Plants. Progress in the Chemistry of Organic Natural Products 108.

[2] Alonso-Castro A.J., Domínguez F., Maldonado-Miranda J.J., Castillo-Pérez L.J., Carranza-Álvarez C., Solano E., Orozco-Castellanos L.M. (2017). Use of medicinal plants by health professionals in Mexico. J. Ethnopharmacol..

[3] Rosas-Piñón Y., Mejía A., Díaz-Ruiz G., Aguilar M.I., Sánchez-Nieto S., Rivero-Cruz J.F. (2012). Ethnobotanical survey and antibacterial activity of plants used in the Altiplane region of Mexico for the treatment of oral cavity infections. J. Ethnopharmacol..

[4] Martínez Ruiz M.G., Gómez-Velasco A., Juárez Z.N., Hernández L.R., Bach H. (2013). Exploring the biological activities of *Echeveria leucotricha*. Nat. Prod. Res..

[5] Reyes V.E. (2023). *Helicobacter pylori* and its role in gastric cancer. Microorganisms.

[6] Castillo-Juárez I., González V., Jaime-Aguilar H., Martínez G., Linares E., Bye R., Romero I. (2009). Anti-*Helicobacter pylori* activity of plants used in Mexican traditional medicine for gastrointestinal disorders. J. Ethnopharmacol..

[7] Palacios-Espinosa J.F., Núñez-Aragón P.N., Gomez-Chang E., Linares E., Bye R., Romero I. (2021). Anti-*Helicobacter pylori* activity of *Artemisia ludoviciana* subsp. mexicana and two of its bioactive components, estafiatin and eupatilin. Molecules.

[8] Solares F., Vázquez-Alvarado J., Gálvez-Cortés M. (2012). Commercialization channels of cuachalalate (*Amphipterygium adstringens* Schiede ex Schlecht.) bark in Mexico. Rev. Mex. Cienc. For..

[9] Galván M.F.V. (2019). El Tronco “Mágico” del Cuachalalate: Regalo de la Medicina Tradicional Mexicana a la Química de Productos Naturales.

[10] Pérez-Contreras C.V., Alvarado-Flores J., Orona-Ortiz A., Balderas-López J.L., Salgado R.M., Zacaula-Juárez N., Navarrete A. (2022). Wound healing activity of the hydroalcoholic extract and the main metabolites of *Amphipterygium adstringens* (*cuachalalate*) in a rat excision model. J. Ethnopharmacol..

[11] Castillo-Juárez I., Rivero-Cruz F., Celis H., Romero I. (2007). Anti-*Helicobacter pylori* activity of anacardic acids from *Amphipterygium adstringens*. J. Ethnopharmacol..

[12] Sotelo-Barrera M., Cília-García M., Luna-Cavazos M., Díaz-Núñez J.L., Romero-Manzanares A., Soto-Hernández R.M., Castillo-Juárez I. (2022). *Amphipterygium adstringens* (Schltdl.) Schiede Ex Standl (Anacardiaceae): An Endemic Plant with Relevant Pharmacological Properties. Plants.

[13] Esquivel-García R., Ayiania M., Abu-Lail N., López-Meza J.E., del Río R.E., García-Pérez M., García-Pérez M.E. (2020). Pyrolytic oils from *Amphipterygium adstringens* bark inhibit IL-8 production of IL-17-stimulated HaCaT keratinocytes. J. Anal. Appl. Pyrolysis.

[14] Gonzalez E.E., Delgado J.N. (1962). Phytochemical investigation of *Amphipterygium adstringens*. J. Pharm. Sci..

[15] Rodríguez-Canales M., Jiménez-Rivas R., Canales-Martínez M.M., García-López A.J., Rivera-Yañez N., Nieto-Yañez O., Rodríguez-Monroy M.A. (2016). Protective effect of *Amphipterygium adstringens* extract on dextran sulphate sodium-induced ulcerative colitis in mice. Mediators Inflamm..

[16] Makino M., Motegi T., Fujimoto Y. (2004). Tirucallane-type triterpenes from *Juliania adstringens*. Phytochemistry.

[17] Díaz-Sánchez L., Zentella-Dehesa A., Castro-Torres V.A., Silva-Jiménez N., Jacobo-Herrera N.J., Martínez-Vázquez M. (2023). Evaluations of anticancer effects of combinations of cisplatin and tirucallane-type triterpenes isolated from *Amphipterygium adstringens* (Schltdl). Chem. Biodivers..

[18] Cofepris (2022). RCC7-Herbolarios. https://www.gob.mx/cms/uploads/attachment/file/777503/RCC7-Herbolarios.PDF.

[19] Comisión Permanente de la Farmacopea de los Estados Unidos Mexicanos (2021). Farmacopea Herbolaria de los Estados Unidos Mexicanos 3.0.

[20] Shaheen S., Ramzan S., Khan F., Ahmad M. (2019). Adulteration in Herbal Drugs: A Burning Issue.

[21] Sharma N., Tiwari N., Vyas M., Khurana N., Muthuraman A., Utreja P. (2020). An overview of therapeutic effects of vanillic acid. Plant Arch..

[22] Kim S.J., Kim M.C., Um J.Y., Hong S.H. (2010). The beneficial effect of vanillic acid on ulcerative colitis. Molecules.

[23] Rainha N., Medeiros V.P., Ferreira C., Raposo A., Leite J.P., Cruz C., Pacheco C.A., Ponte D., Silva A.B. (2016). Leaf malate and succinate accumulation are out of phase throughout the development of the CAM plant *Ananas comosus*. Plant Physiol. Biochem..

[24] Zhuoma Y., Yang M., Chen Y., Zhang X., Duan X., Cui H., Hu X. (2025). NMR-Based Metabolomics Analysis of Metabolite Profiles in Two Species of Boletes Subjected to Different Drying Methods. Metabolites.

[25] Ochoa-Jiménez V.A., Berumen-Varela G., Pérez-Ramírez I.F., Balois-Morales R., Rubio-Melgarejo A., Bautista-Rosales P.U. (2024). Metabolomics approach for phenolic compounds profiling of soursop (*Annona muricata* L.) fruit during postharvest storage. Metabolomics.

[26] Chen Z., Dai W., Xiong M., Gao J., Zhou H., Chen D., Li Y. (2024). Metabolomics investigation of the chemical variations in white teas with different producing areas and storage durations. Food Chem. X.

[27] Gómez-Salgado M.D.R.H., Beltrán-Gómez J.Á., Díaz-Núñez J.L., Rivera-Chávez J.A., García-Contreras R., Estrada-Velasco Á.Y., Castillo-Juárez I. (2024). Efficacy of a Mexican folk remedy containing cuachalalate (*Amphipterygium adstringens* (Schltdl.) Schiede ex Standl) for the treatment of burn wounds infected with *Pseudomonas aeruginosa*. J. Ethnopharmacol..

[28] Arenas-Quevedo M.G., Gracia-Fadrique J. (2024). *Amphipterygium adstringens* (cuachalalate) extract by supercritical CO_2_. Chem. Thermodyn. Therm. Anal..

