# Supplementary material for: 1H Nuclear Magnetic Resonance-Based Targeted and Untargeted Metabolomics Profiling of Retail Samples of Cuachalalate (Amphipterygium adstringens)
Source: Molecules. 2025 May 16;30(10):2185. doi: 10.3390/molecules30102185 (PMC12113676; doi:10.3390/molecules30102185)
Supplement: Supplementary file 1 [file molecules-30-02185-s001.zip › molecules-3563361-supplementary.pdf]

^{1}H NMR-based targeted and untargeted metabolomic profiling of retail samples of cuachalalate (*Amphipterygium adstringens*)

Erick Alejandro Herrera-Jurado [1], Estefanía De Jesús Terán-Sánchez [1], José Iván Serrano-Contreras [2] and L. Gerardo Zepeda-Vallejo [1,*]

[1] Departamento de Química Orgánica, Escuela Nacional de Ciencias Biológicas, Instituto Politécnico Nacional, Prolongación de Carpio y Plan de Ayala s/n, Col. Santo Tomas., Delegación Miguel Hidalgo, Mexico City C.P. 11340, Mexico; erick.cqb@gmail.com (E.A.H.-J.); estefaniateran92@gmail.com (E.D.J.T.-S.)

[2] Department of Metabolism, Digestion and Reproduction, Section of Biomolecular Medicine, Faculty of Medicine, Imperial College London, South Kensington Campus, London SW7 2AZ, UK; j.serrano-contreras@imperial.ac.uk

* Correspondence: lzepeda@ipn.mx; Tel.: +52-55-5729-6300 (ext. 62412)

Figure S1. ^{1}H NMR spectra of the selective extracts of 3a-hidroxymasticadienoic acid (3α-HMDA) obtained from cuachalalate in different organic solvents: A) Trichloroethylene, B) Dichloromethane, C) Hexane, D) Acetonitrile. Signals intensities are referenced to the signal of 10 mM hexamethyldisilane in $CHCl_3$.

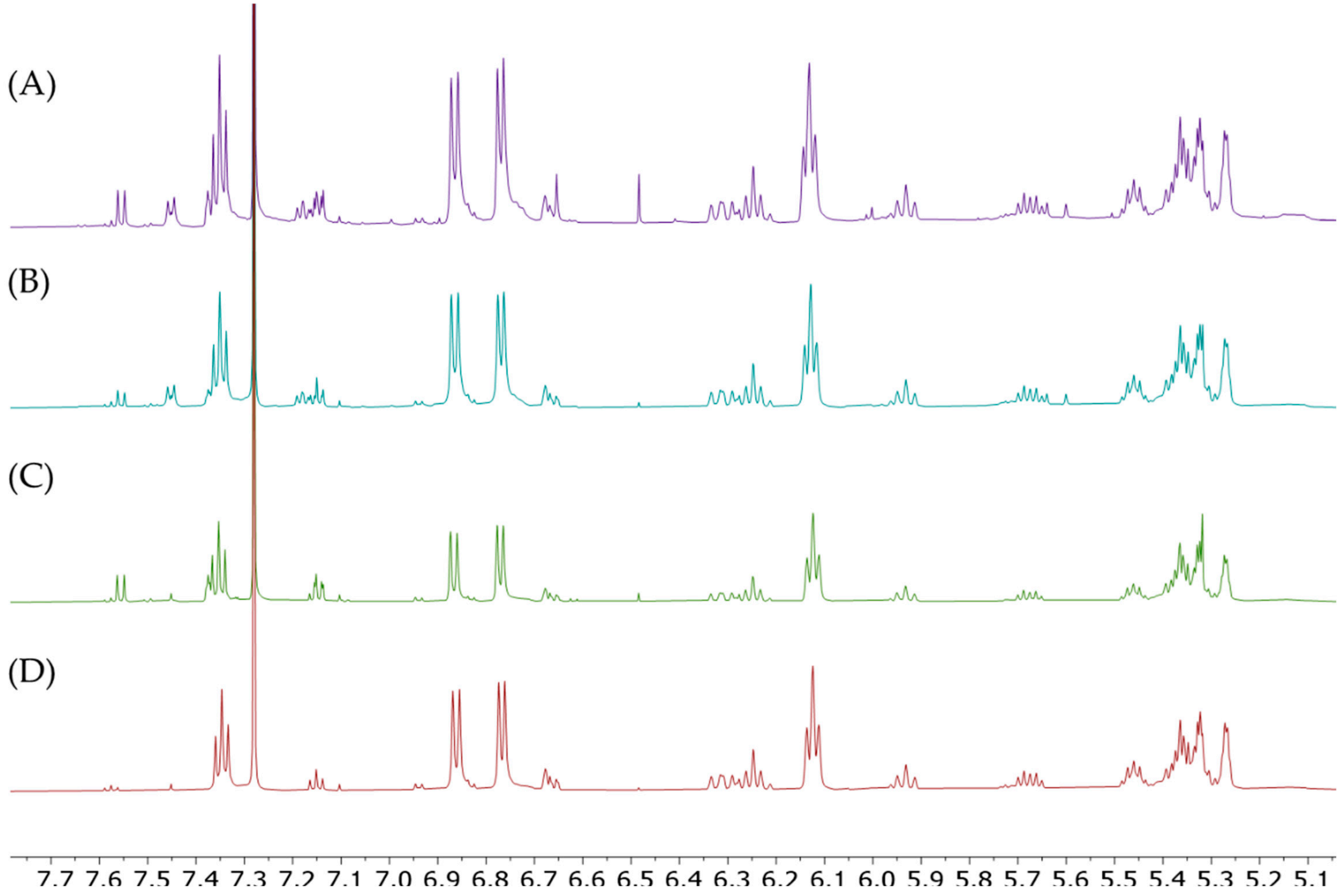

Figure S2. ^{1}H and ^{13}C NMR data of 3α-hydroxymasticadienoic acid (3α-HMDA). The NMR data described below were obtained on a Bruker Avance III 600 MHz spectrometer. The corresponding multiplicities, coupling constants, and assignments were performed by using the series of 1D and 2D NMR experiments shown in Figures S3-S7. The analyzed sample was obtained after separation through a column chromatography packed with 300-400 mesh silica gel, using a 3:2 hexane:ethyl acetate mixture as eluent.

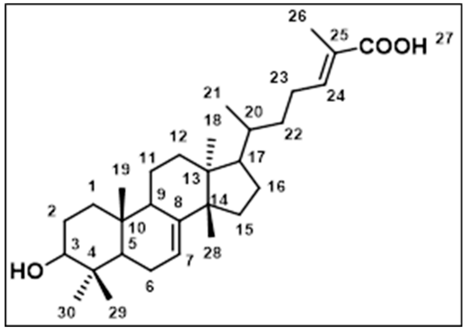

RMN ^{1}H ($CDCl_3$, 600.18 MHz):

δ 6.00 (1H, t, J = 7.4 Hz, H24), 5.23 (1H, bs, H7), 3.45 (1H, m, H3), 2.53 (m, H23a), 2.41 (m, H23b), 2.32 (m, H9), 2.04 (m, H12a), 1.94 (1H, m, H16a), 1.93 (1H, m, H12b), 1.92 (1H, m, H2a), 1.9 (3H, bs, H26), 1.77 (1H, m, 6a), 1.75 (1H, m, H5), 1.61 (1H, m, H2b), 1.61 (2H, m, H6b), 1.54 (1H, m, H11a), 1.52 (1H, m, H22a), 1.49 (1H, m, H15a), 1.48 (1H, m, H17), 1.47 (1H, m, H11b), 1.45 (1H, m, H1a), 1.44 (1H, m, H15b), 1.4 (1H, m, H20), 1.38 (1H, m, H1b), 1.26 (1H, m, H16b), 1.12 (1H, m, H22b), 0.97 (3H, s, H28), 0.94 (3H, s, H30), 0.91 (3H, s, H29), 0.88 (3H, d, J = 6.42 Hz, H21), 0.81 (3H, s, H18), 0.76 (3H, s, H19).

RMN ^{13}C ($CDCl_3$, 150.93 MHz):

δ 176.1 (C27), 146.1 (C8), 145.5 (C24), 126.2 (C25), 117.8 (C7), 76.2 (C3), 52.8 (C17), 51.2 (C14), 48.6 (C9), 44.5 (C5), 43.48 (C4), 37.3 (C13), 36.0 (C20), 35.7 (C22), 34.7 (C10), 33.9 (C15), 33.7 (C6), 31.2 (C1), 28.2 (C16), 27.7 (C30), 27.2 (C28), 26.8 (C23), 25.3 (C2), 23.9 (C12), 21.8 (C18), 21.8 (C29), 20.6 (C26), 18.2 (C21), 17.9 (C11), 12.9 (C19).

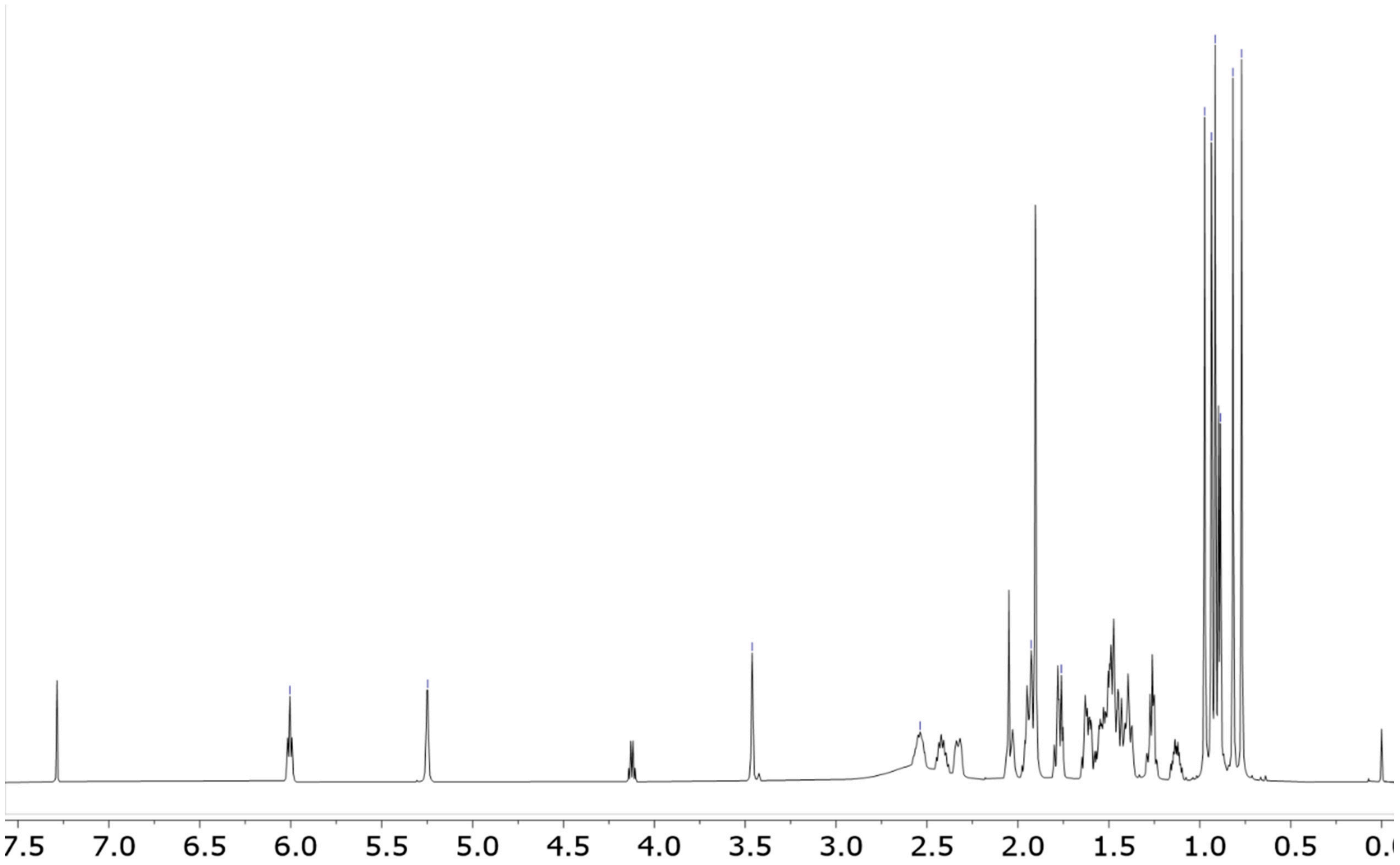

Figure S3. ^{1}H NMR spectrum of the purified biomarker 3α-HMDA.

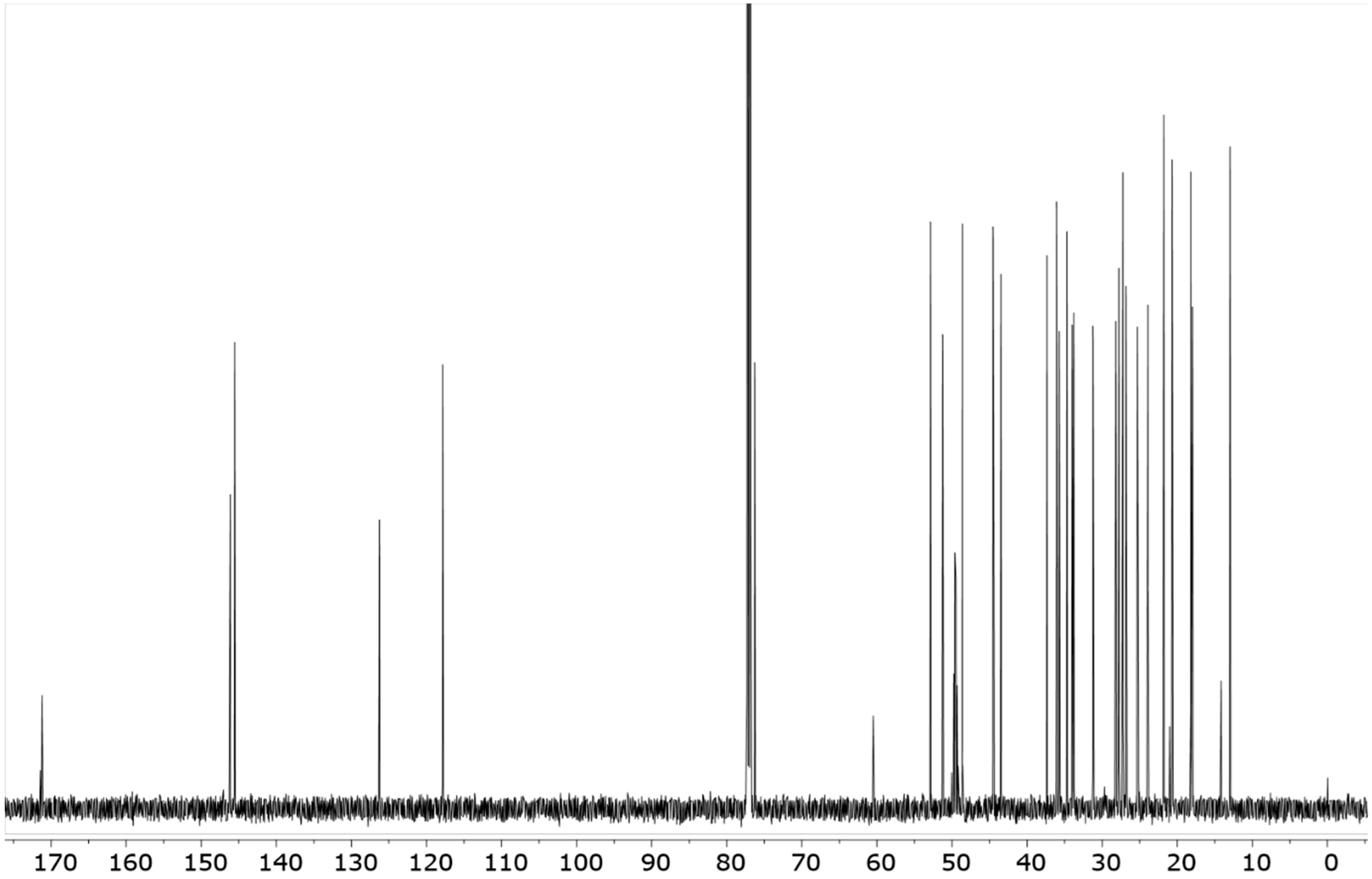

Figure S4. ^{13}C NMR spectrum of the purified biomarker 3αHMDA ($CDCl_3$, 150 MHz).

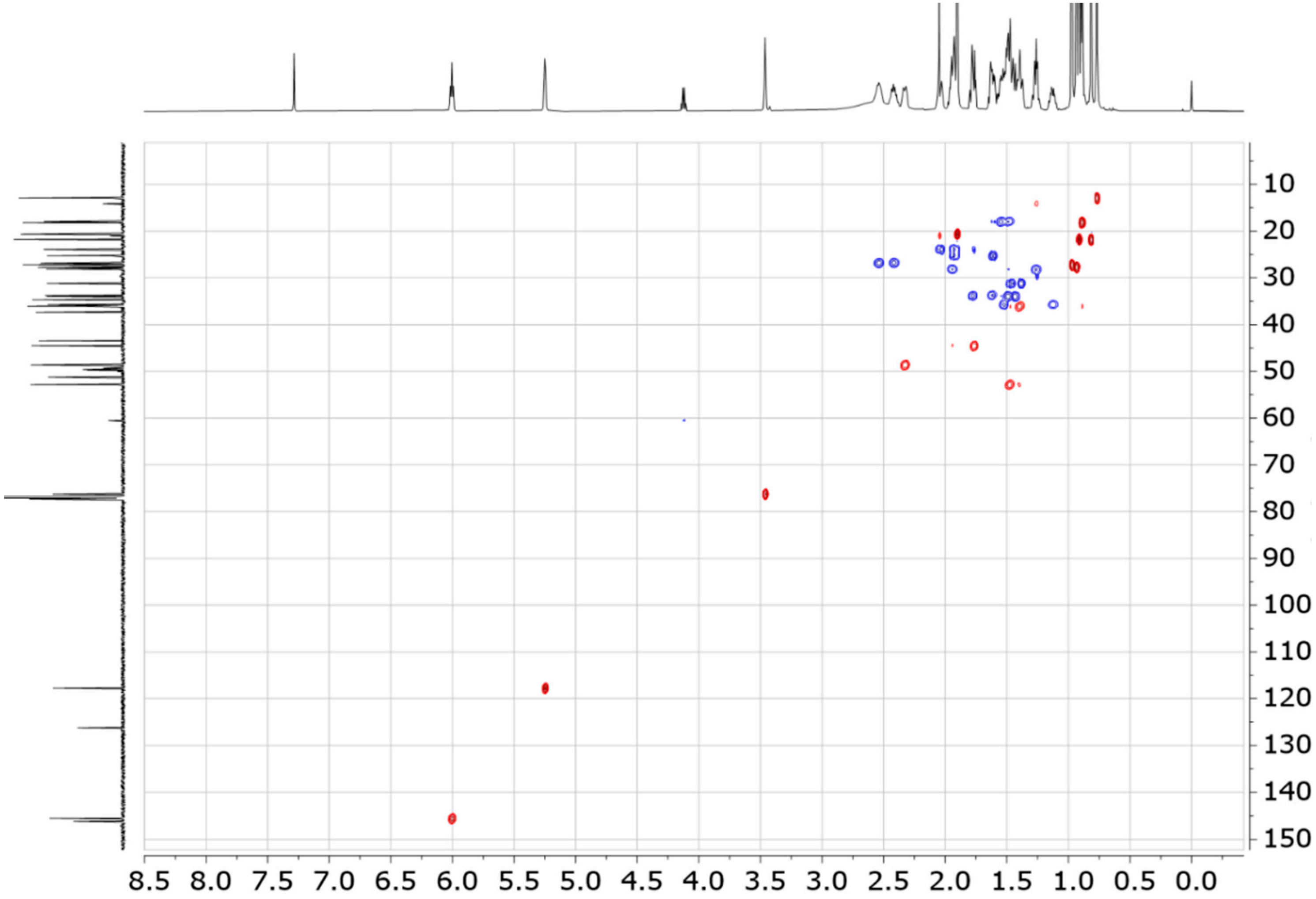

Figure S5a. ^1H-^{13}C HSQC spectrum of the purified biomarker 3α-HMDA ($CDCl_3$, 600 MHz).

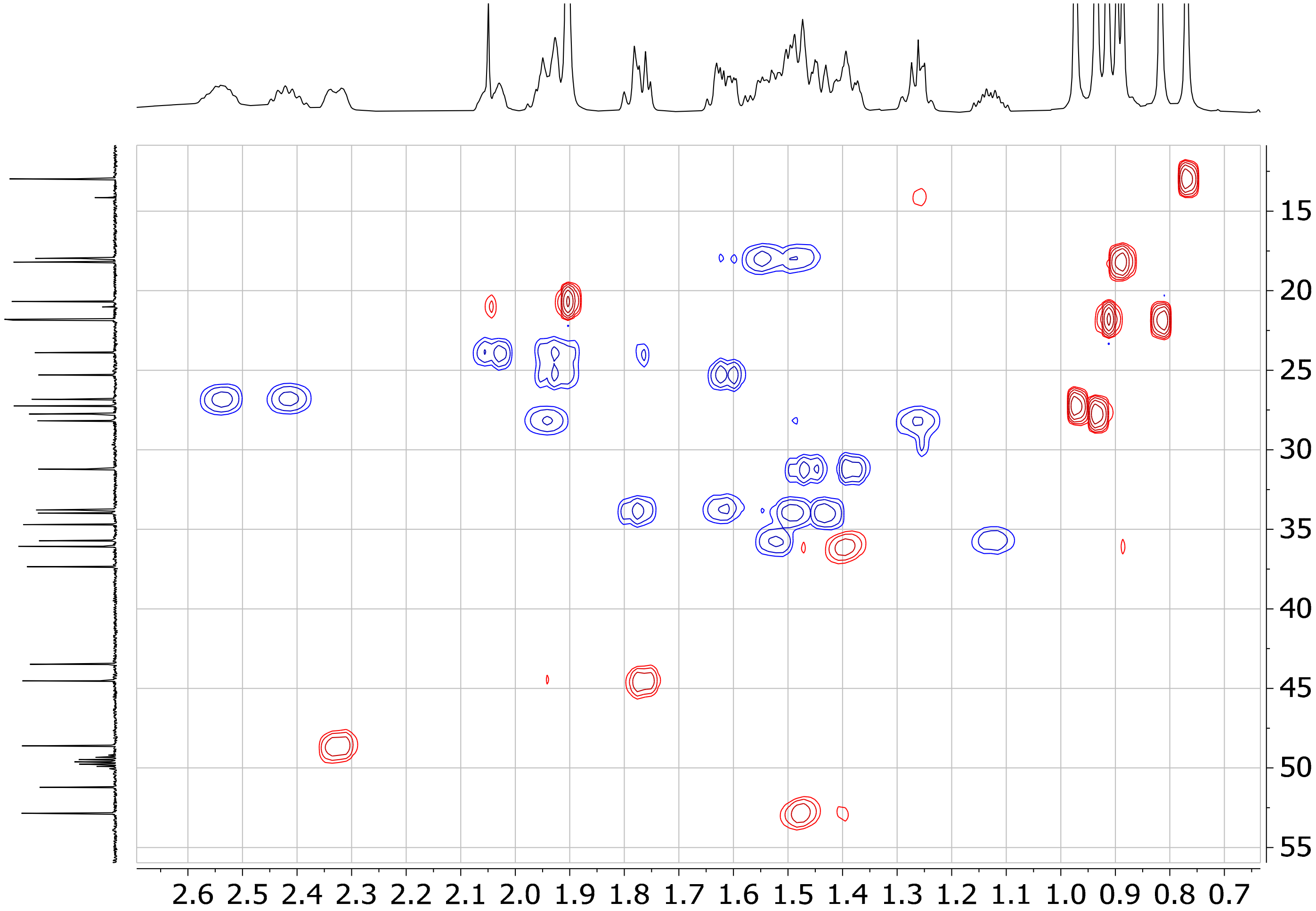

Figure S5b. Expanded region of the ^1H-^{13}C HSQC spectrum of 3α-HMDA (CDCl$_3$, 600 MHz).

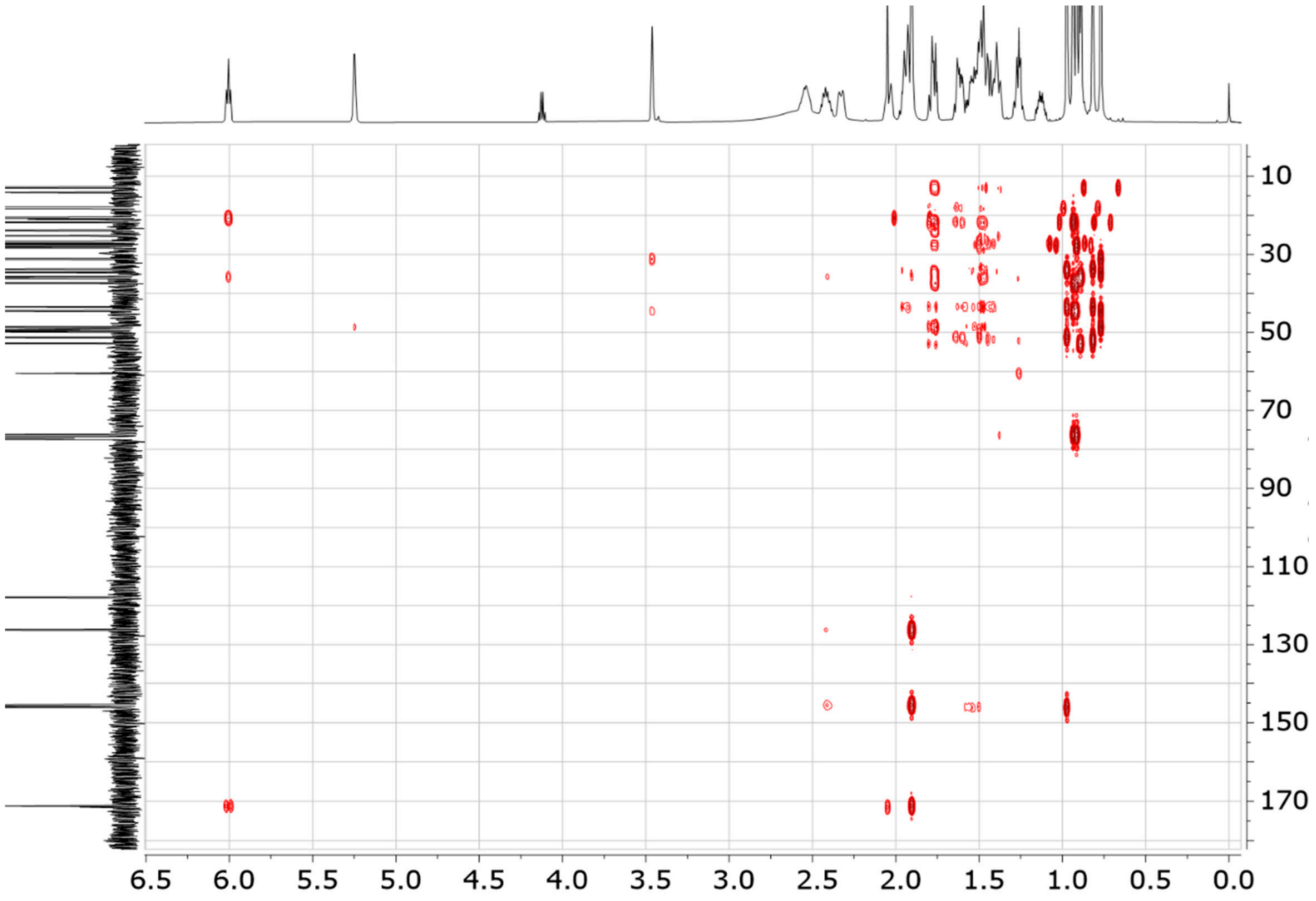

Figure S6a. HMBC spectrum of the purified biomarker 3α-HMDA ($CDCl_3$, 600 MHz).

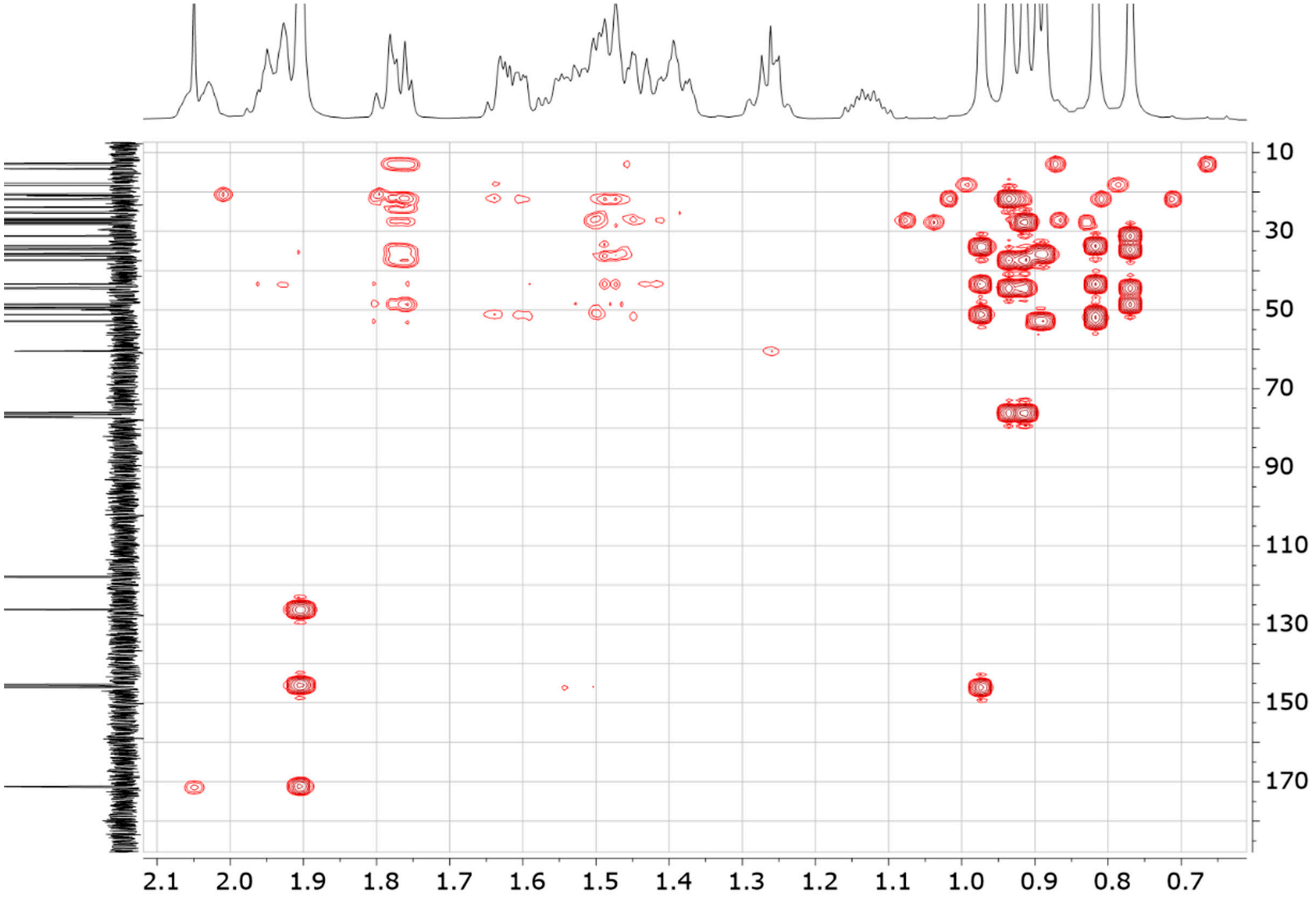

Figure S6b. Expanded region of the HMBC spectrum of 3α-HMDA ($CDCl_3$, 600 MHz).

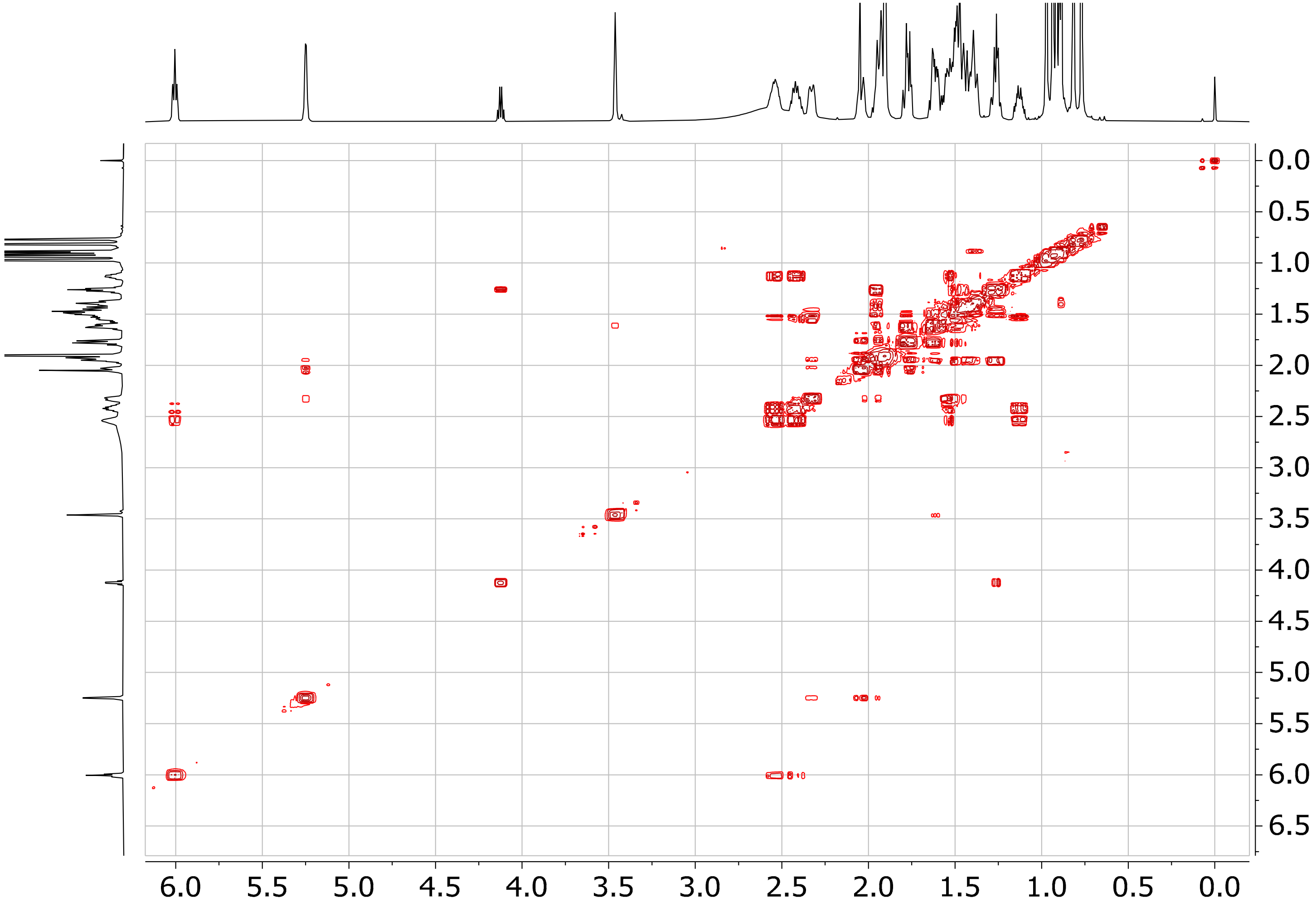

Figure S7. ^1H-^1H COSY spectrum of the purified biomarker 3α-HMDA ($CDCl_3$, 600 MHz).

Figure S8. PCA (A) and PLS-DA (B) score plots of all samples including QC, labeled as SMP11.

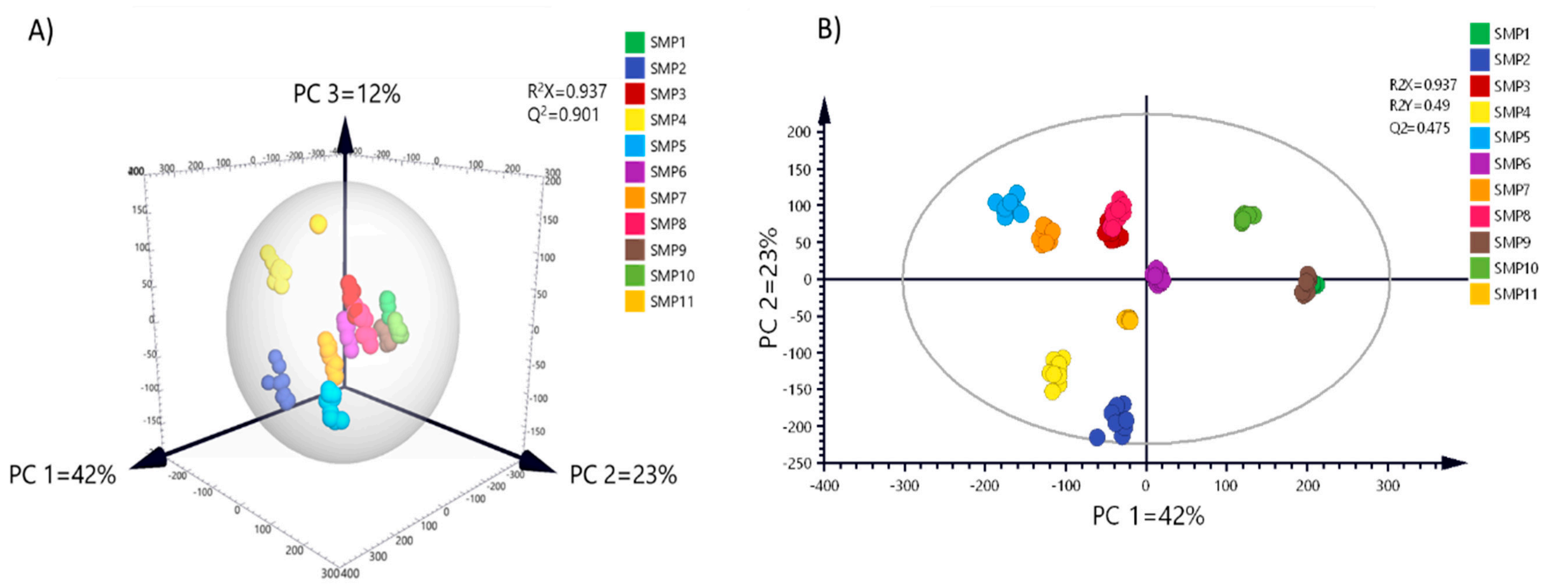

Figure S9. Bar graphs show the relative concentration of the identified metabolites in the aqueous extract. ANOVA (t-student, p = 0.05).

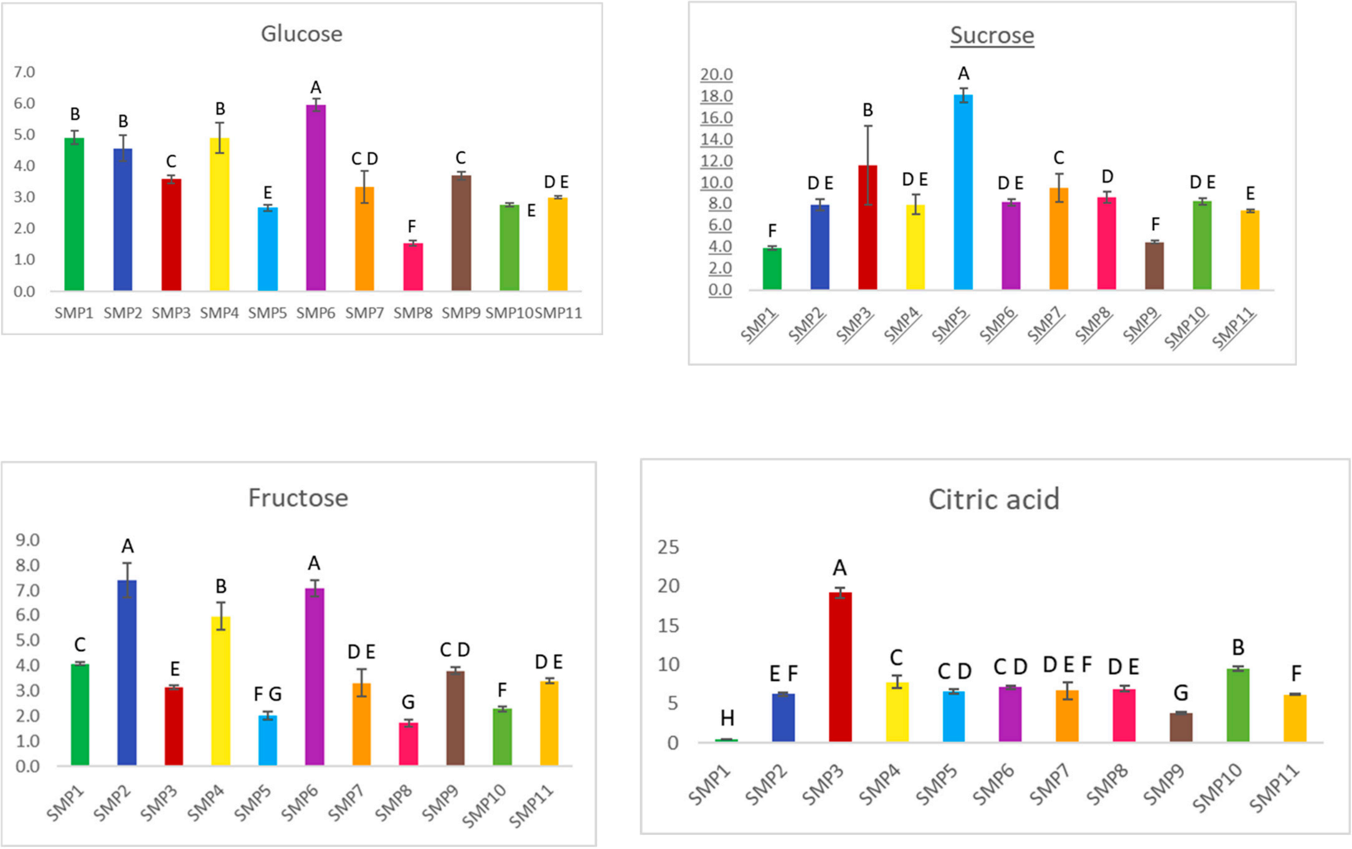

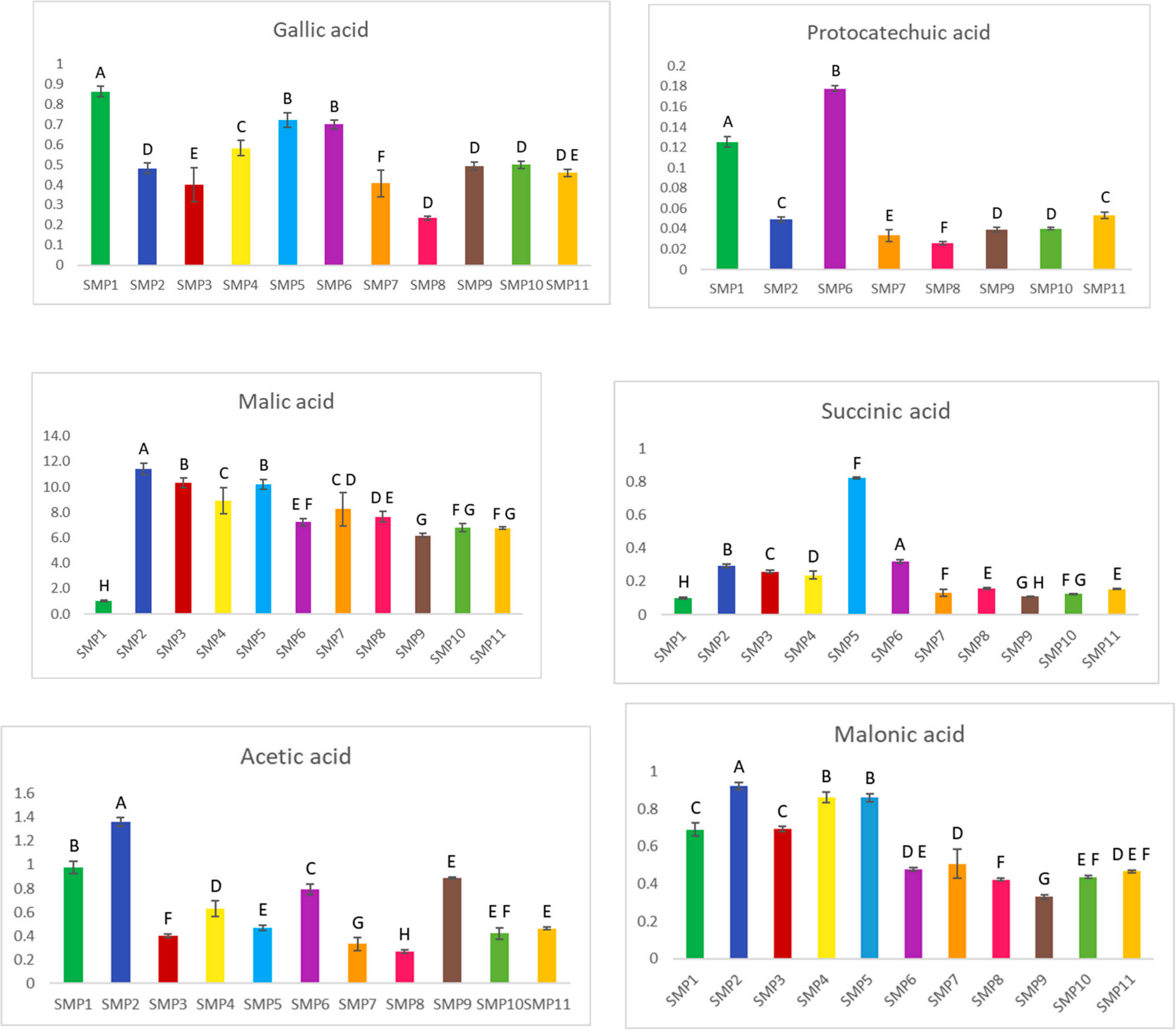
Gallic acid
1
0.9
0.8
0.7
0.6
0.5
0.4
0.3
0.2
0.1
0
A D E C B B F D D D D E
SMP1 SMP2 SMP3 SMP4 SMP5 SMP6 SMP7 SMP8 SMP9 SMP10 SMP11
Protocatechuic acid
0.2
0.18
0.16
0.14
0.12
0.1
0.08
0.06
0.04
0.02
0
A C B E F D D C
SMP1 SMP2 SMP6 SMP7 SMP8 SMP9 SMP10 SMP11
Malic acid
14.0
12.0
10.0
8.0
6.0
4.0
2.0
0.0
H A B C B E F C D D E G F G F G
SMP1 SMP2 SMP3 SMP4 SMP5 SMP6 SMP7 SMP8 SMP9 SMP10 SMP11
Succinic acid
1
0.8
0.6
0.4
0.2
0
H B C D F A F E G H F G E
SMP1 SMP2 SMP3 SMP4 SMP5 SMP6 SMP7 SMP8 SMP9 SMP10 SMP11
Acetic acid
1.6
1.4
1.2
1
0.8
0.6
0.4
0.2
0
B A F D E C G H E E F E
SMP1 SMP2 SMP3 SMP4 SMP5 SMP6 SMP7 SMP8 SMP9 SMP10 SMP11
Malonic acid
1
0.8
0.6
0.4
0.2
0
C A C B B D E D F G E F D E F
SMP1 SMP2 SMP3 SMP4 SMP5 SMP6 SMP7 SMP8 SMP9 SMP10 SMP11

Figure S10a. ^{1}H NMR signals of protocatechuic acid identified with Chenomx® in SMP1.

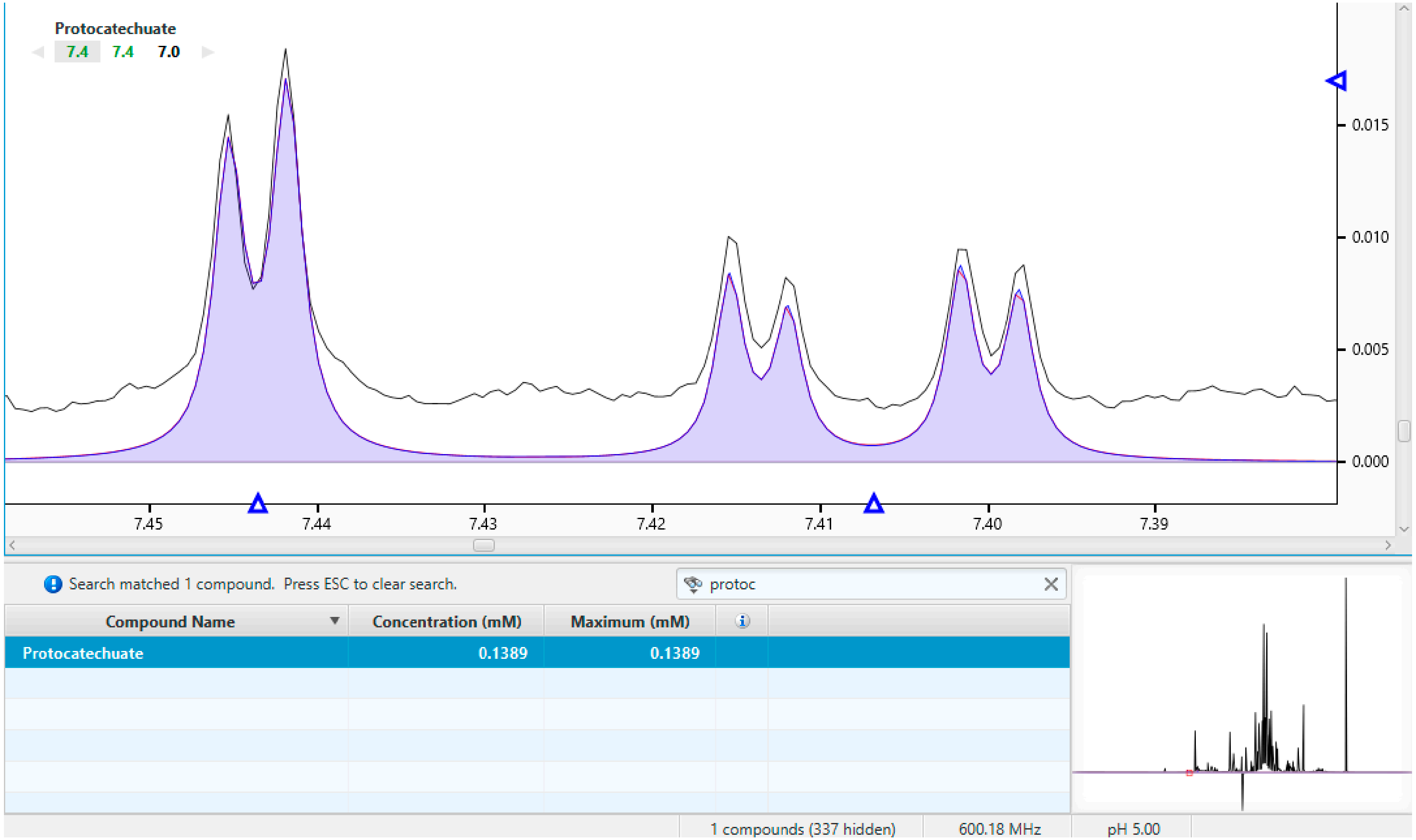

Figure S10b. ^{1}H NMR spectrum of the aqueous extract of cuachalalate showing signals of protocatechuic acid in SMP1. The ^{1}H NMR signals for aromatic protons (7.3-7.5 ppm) in the theoretical spectrum match very well those of the experimental spectrum.

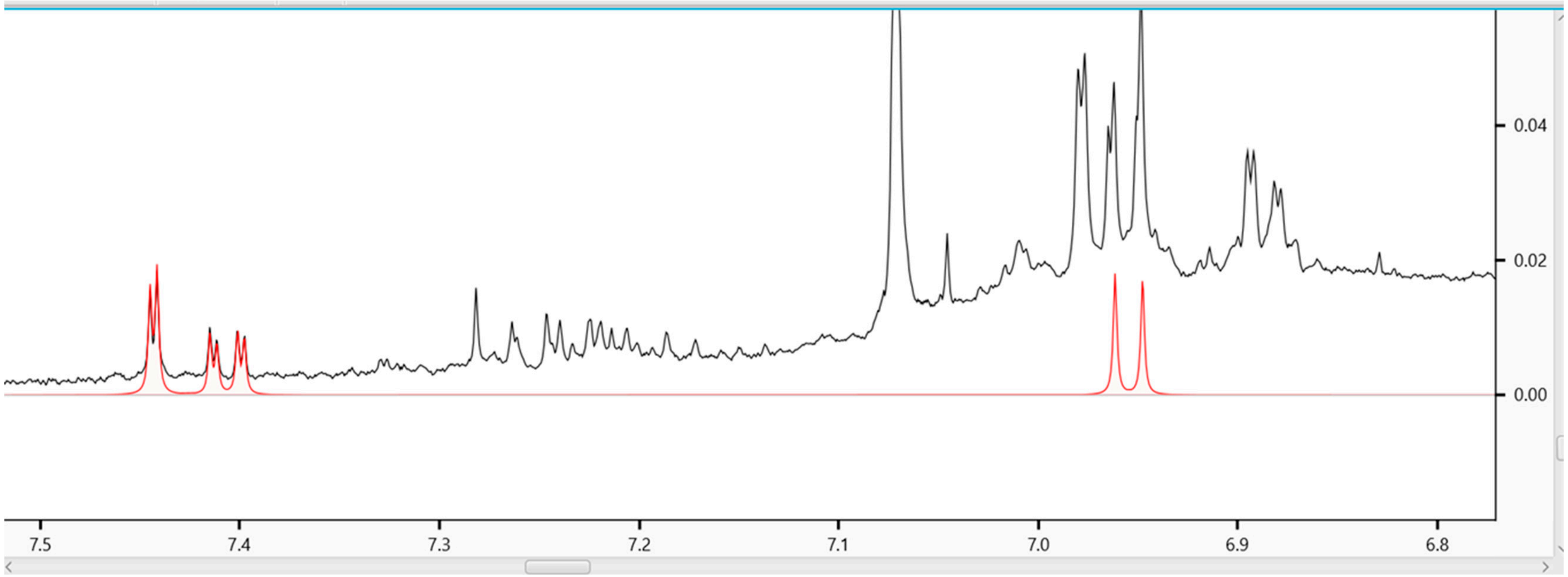

Figure S10c. ^{1}H NMR spectrum of the aqueous extract of cuachalalate (SMP4) showing very weak signals of protocatechuic acid. Similar behavior was observed for samples SMP2, SMP3, SMP5-SMP10. It can be observed that the metabolite signals are negligible due to their low concentration.

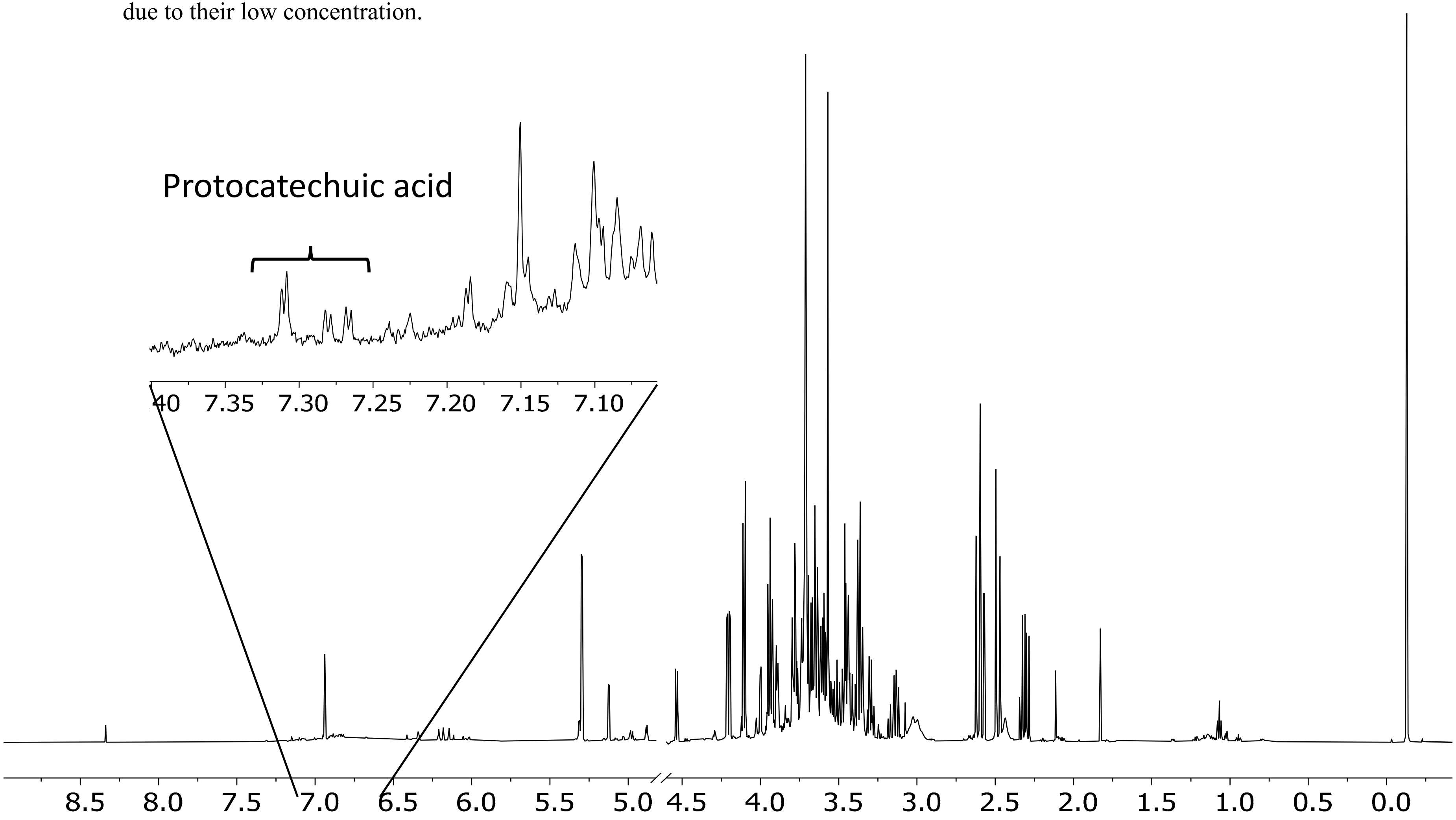

Figure S11. PCA of aqueous matrix of SMP1-SMP10 A) including sugars (MIS) and B) excluding sugars (MES).

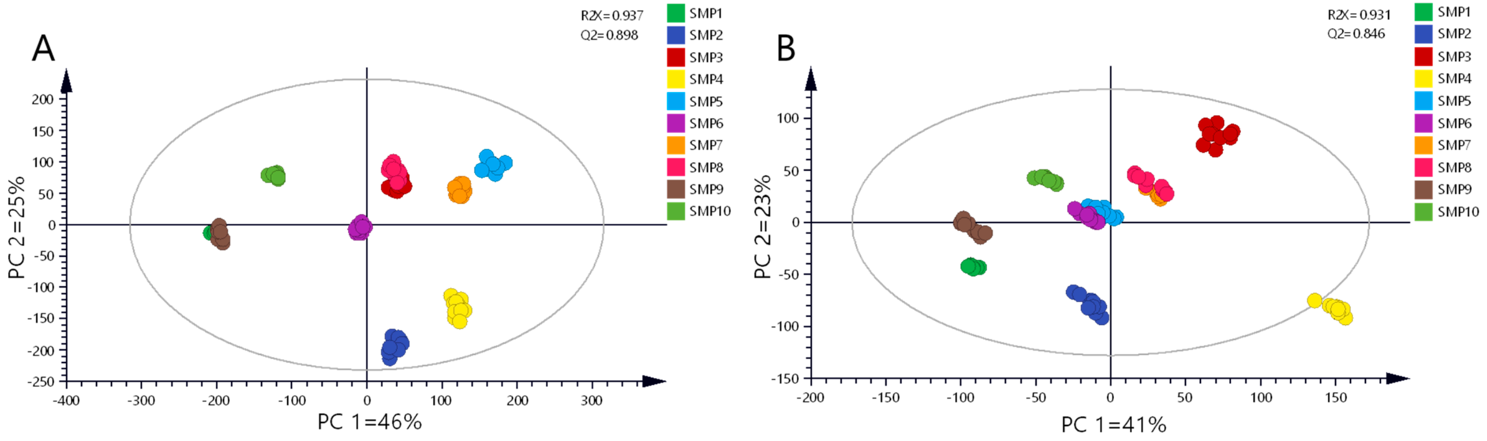

Figure S12. Validation of the PLS-DA model on MIS (top) and MES (bottom) samples using 200 permutations.

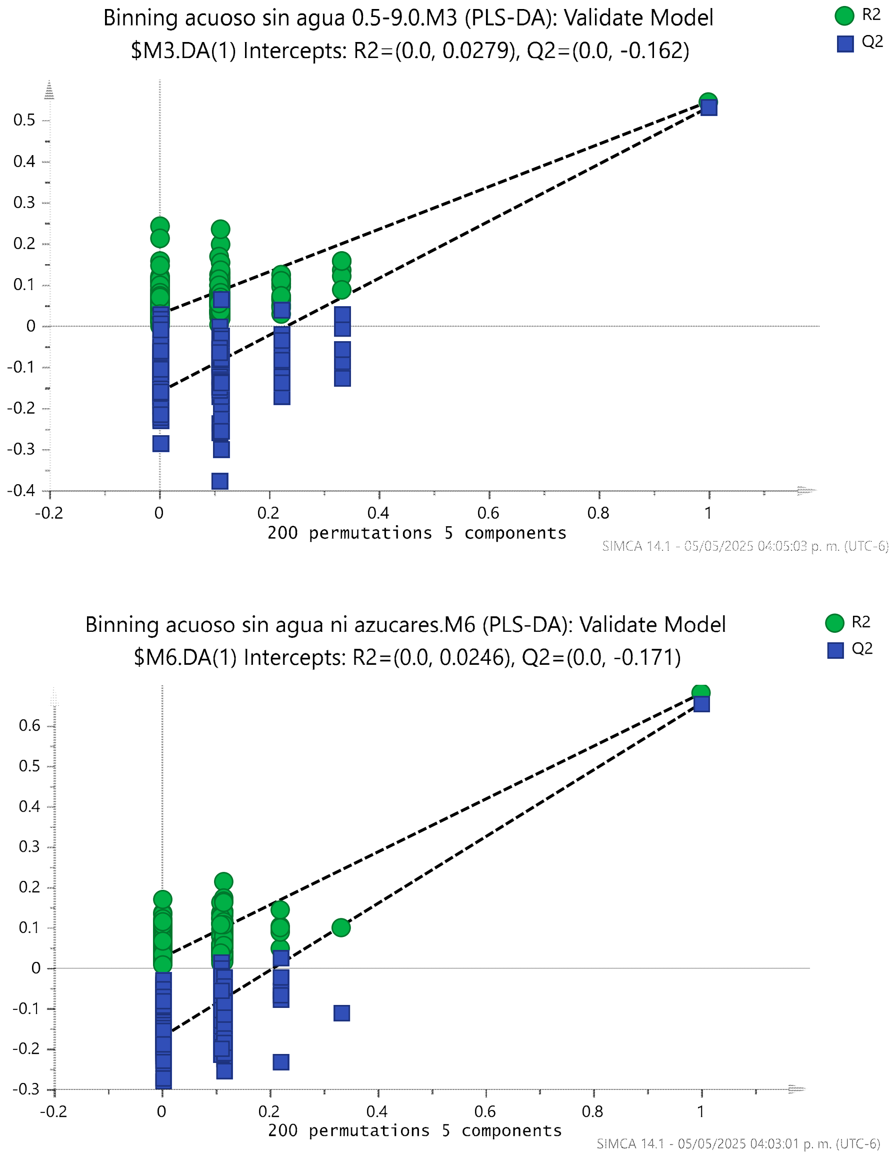

Figure S13. Validation of the PLS-DA model on MIS (top) and MES (bottom) samples using 200 permutations.

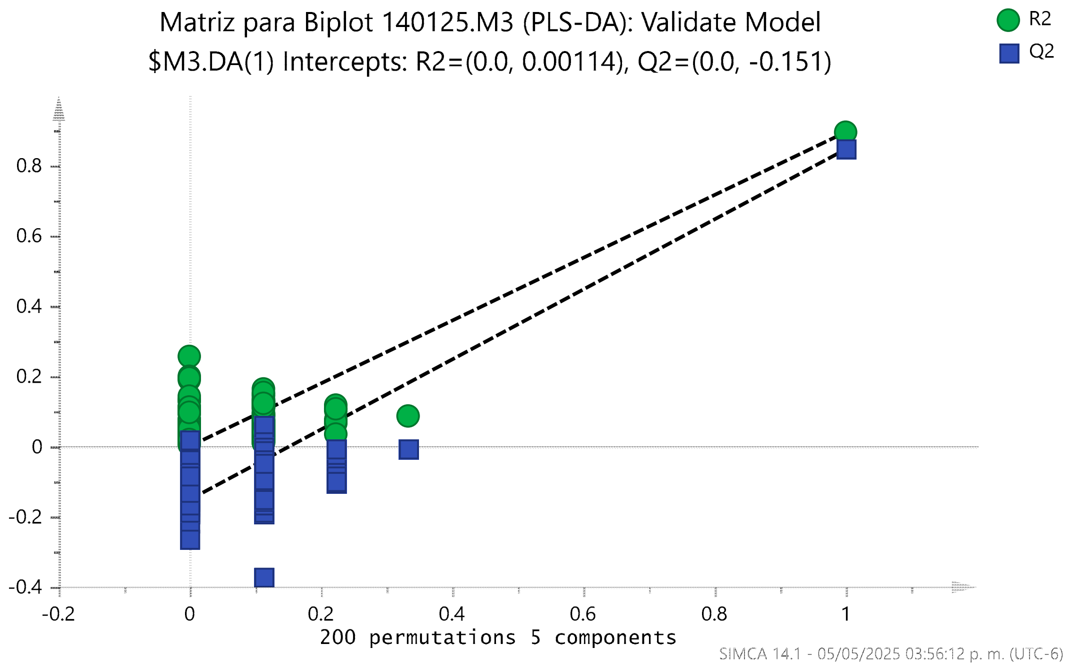

Figure S14. Multivariate statistics on organic extracts. A) PCA, B) PLS-DA, C) Loading plot and D) S-plot.

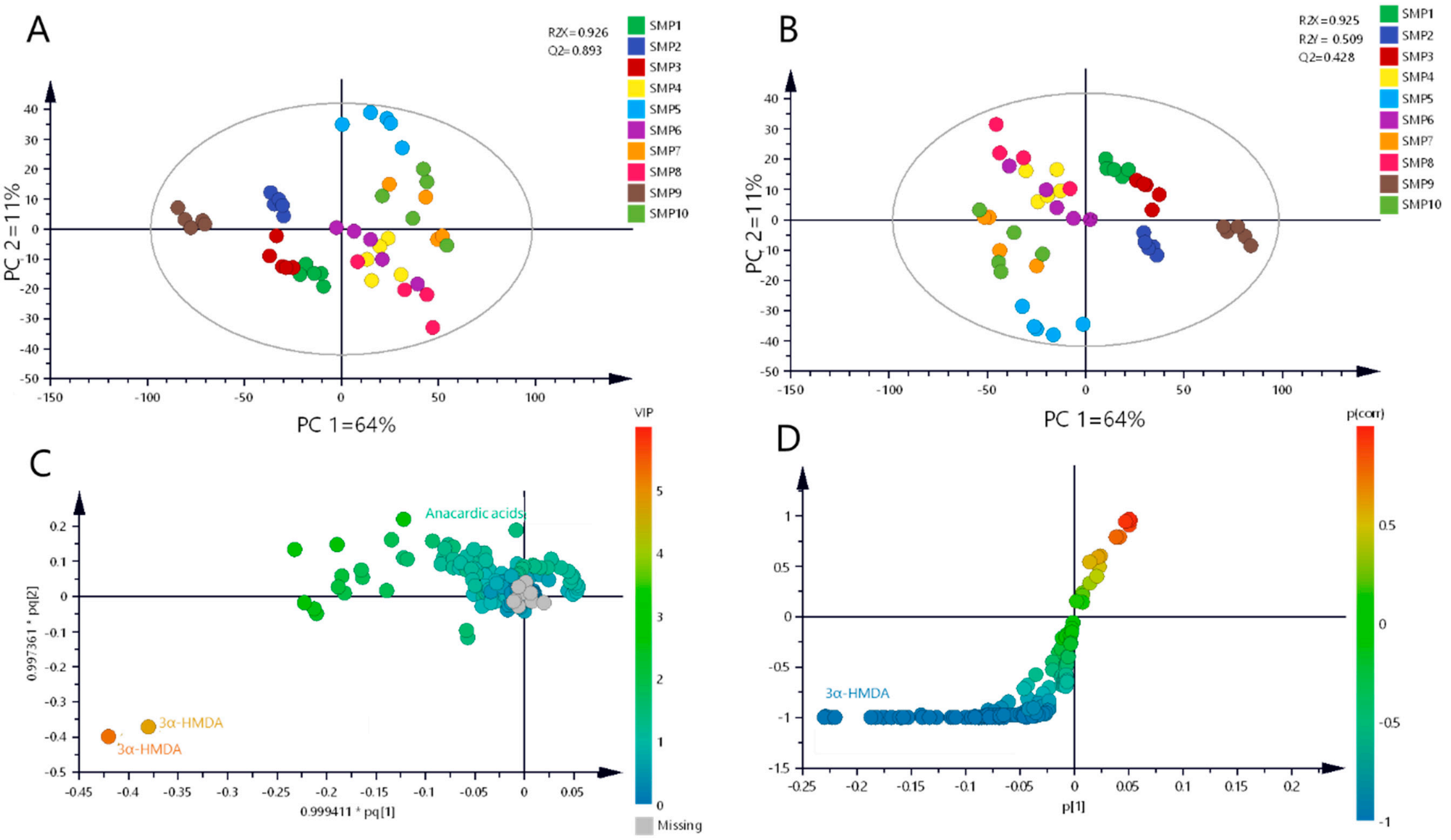

Figure S15. ^{1}H NMR spectrum of anacardic acids isolated from cuachalalate. Expansion region shows in detail signals belonging to aromatic protons ($CDCl_3$, 600 MHz).

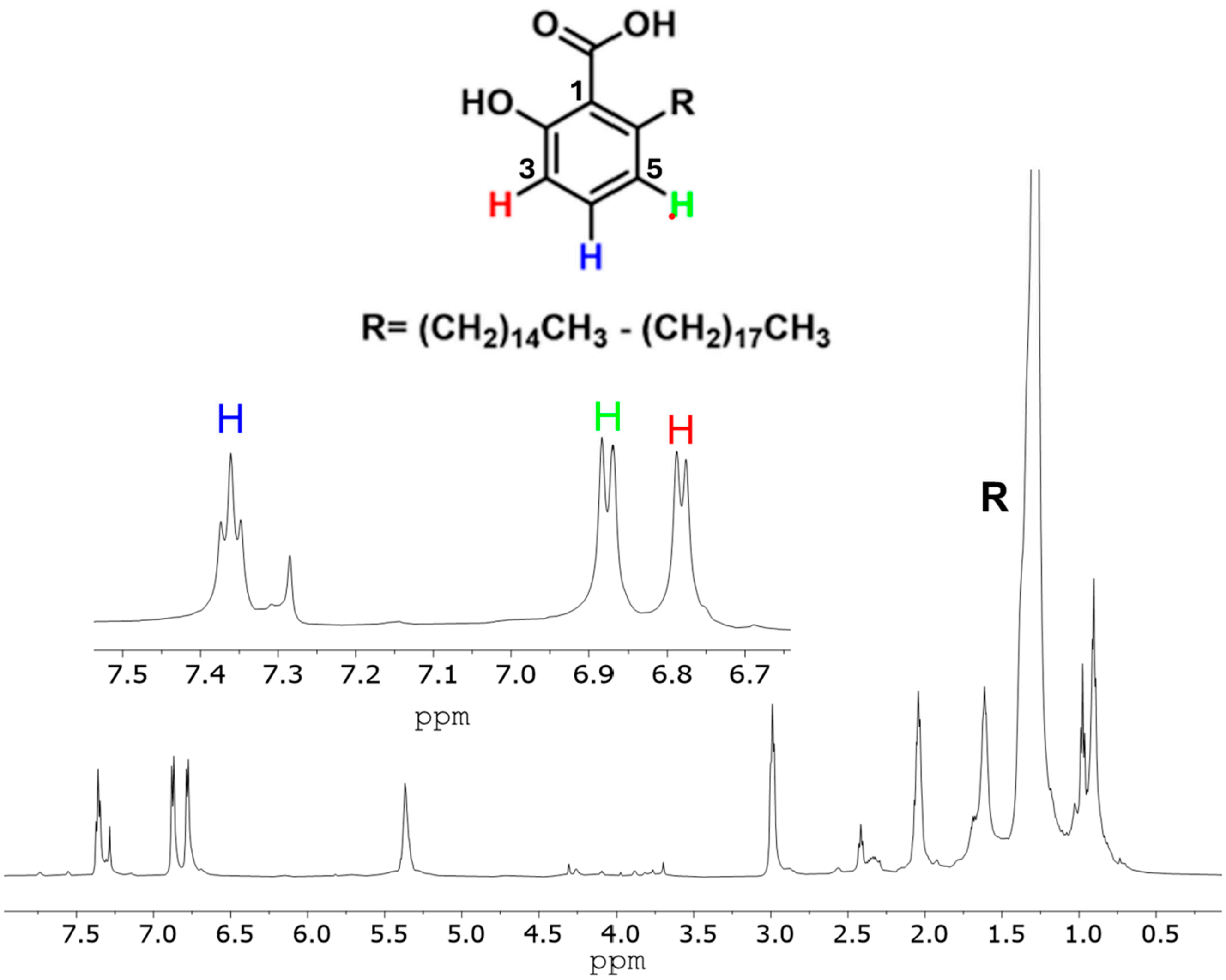

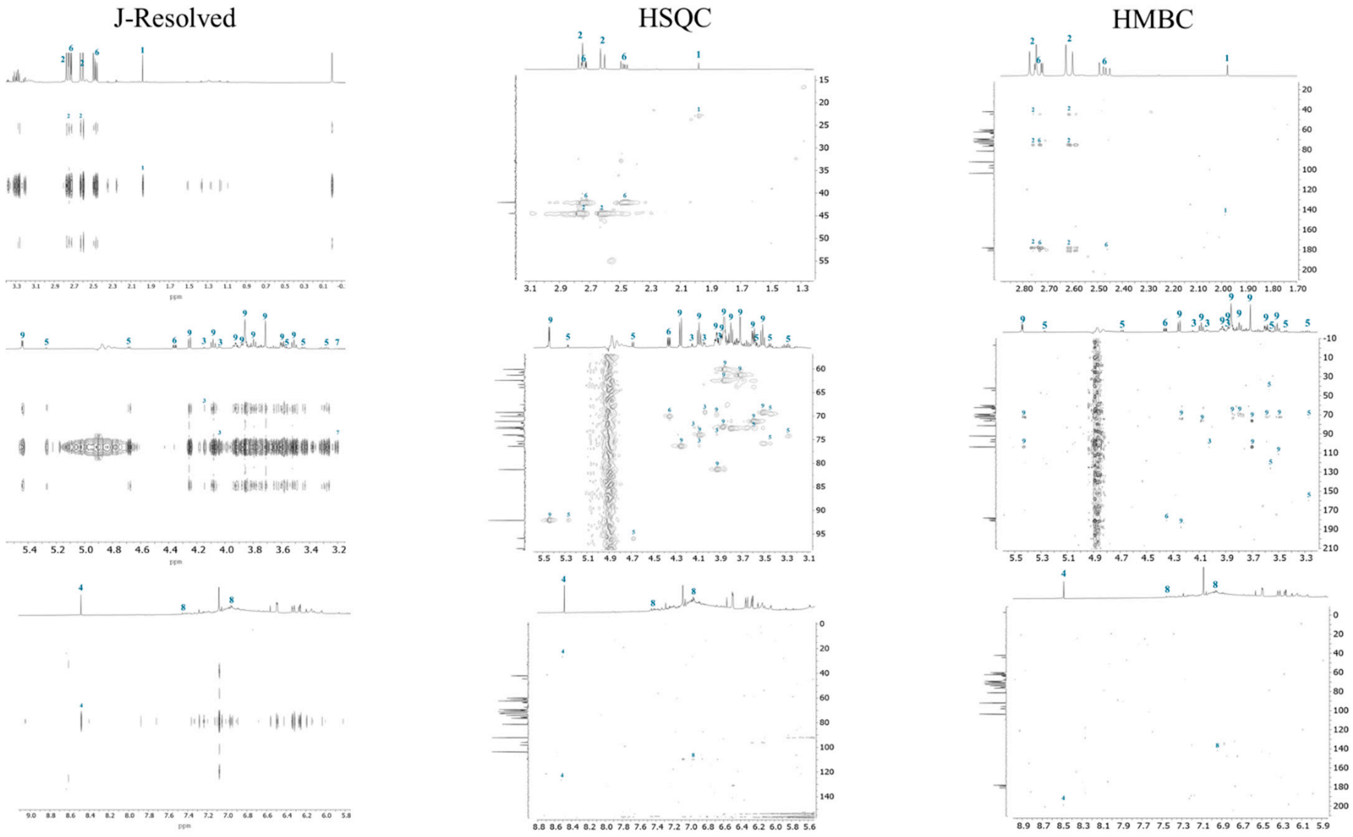

Figure S16. 2D NMR spectra of cuachalalate aqueous extract (H_2O:D_2O 90:10, 600 MHz) used to confirm metabolite identification. Once the metabolites present in the aqueous mixture were identified by Chenomx®, they were confirmed by the set of spectra shown: J-resolved, allowed accurate determination of chemical shifts and scalar coupling in partially overlapping signals; 1H-1H COSY, provided connectivity information via scalar coupling; HSQC, allowed assignment of carbons associated with each proton via heteronuclear scalar coupling to one bond; HMBC, was useful for assignment of carbons exhibiting heteronuclear scalar couplings to two and three bonds.

Table S1. The samples were obtained at their points of sale, processed to a powdered consistency, and stored in resealable bags in a dry and dark place until analysis.

SAMPLE	POINTS OF SALE (OR COLLECT, SMP1)
SMP1	La Capilla, Arteaga, Michoacán; 18°20’09.3’’N, 102°17’13.3’’W
SMP2	Calzada México-Tacuba Popotla, 11400 Ciudad de México, CDMX
SMP3	Balderas 33, Colonia Centro, Centro, Cuauhtémoc, 06040 Ciudad de México, CDMX
SMP4	Calz. de Tlalpan 1549, Portales Nte, Benito Juárez, 03330 Ciudad de México, CDMX
SMP5	Abasolo, CEDA, Iztapalapa, 09040 Ciudad de México, CDMX
SMP6	Abasolo, CEDA, Iztapalapa, 09040 Ciudad de México, CDMX
SMP7	Circunvalación, La Merced, Zona Centro, Venustiano Carranza, 15100 Ciudad de México
SMP8	Circunvalación, La Merced, Zona Centro, Venustiano Carranza, 15100 Ciudad de México
SMP9	Fray Servando Teresa de Mier 419, Merced Balbuena, Venustiano Carranza, 15810 Ciudad de México, CDMX
SMP10	Fray Servando Teresa de Mier 419, Merced Balbuena, Venustiano Carranza, 15810 Ciudad de México, CDMX

Table S2. Identified metabolites in the aqueous extract of retail samples of cuachalalate.

No.	Metabolite	Structure	Assignment	^{1}H	^{13}C
1	Acetic acid	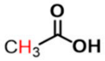	CH3	1.95 (s)	26.1
					188.6
2	Citric acid	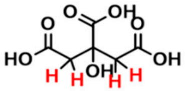	$2CH_2$	2.73 (d, 15.4 Hz)	44.5
				2.61 (d, 15.4 Hz)	76.5
					178.1
					181.4
					178.2
3	Fructose	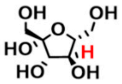	CH	4.11 (d, 3.7 Hz)	70.1
			→ CH	4.02 (dd, 12.9, 1.0 Hz)	76.5
			CH_2	3.90 (dd, 11.3, 5.8 Hz)	74.2
				3.80 (d, 9.7 Hz)	
			CH_2	3.72 (d, 11.3 Hz)	81.4
			CH	3.60 (d, 12.1 Hz)	
			CH	3.56 (d, 11.6 Hz)	72.8
4	Gallic acid	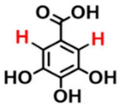	2 CH	7.0 (s)	109.8
					160.5
5	α,ß-Glucose	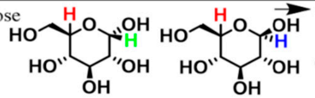	→ CH	5.24 (d, 3.7 Hz)	92.5
				3.54 (dd, 9.4, 3.5 Hz)	72.5
			CH	4.65 (d, 7.9 Hz)	
				3.57 (t, 10.3 Hz)	72.7
				3.42 (t, 9.4 Hz)	71.1
				3.40 (t, 9.4 Hz)	72.4
			CH	3.25 (dd, 9.4, 8.2 Hz)	69.3
6	Malic acid	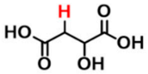			178.2
			CH_2	2.70 (dd, 15.3, 3.3 Hz)	42.2
				2.42 (dd, 15.3, 9.5 Hz)	42.2
7	Malonic acid	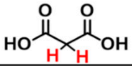	CH_2	3.20 (s)	
					162.6
8	Protocatechuic acid	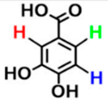	CH	7.39 (dd, 8.2, 2.0 Hz)	
			→ CH	7.43 (d, 2.0 Hz)	
			CH	6.94 (d, 8.2 Hz)	
9	Sucrose	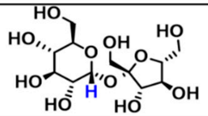	CH	5.41 (d, 3.7 Hz)	92.2
				4.23 (d, 8.5 Hz)	104.3
				5.41 (d, 3.7 Hz)	
10	Succinic acid	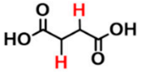	2 CH_2	2.47 (s)	41.9
					180.1
11	Vanillic acid	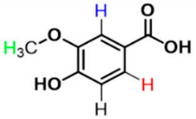	→ CH	7.44 (d, 1.8 Hz)	41.9
			CH	7.40 (dd, 8.4, 1.8 Hz)	180.1
			CH	6.95 (d, 8.4 Hz)	
			CH_3	3.90 (s)	
